# Supplementary material for: Characterization and structure-based protein engineering of a regiospecific saponin acetyltransferase from Astragalus membranaceus
Source: Nat Commun. 2023 Sep 25;14:5969. doi: 10.1038/s41467-023-41599-7 (PMC10519980; doi:10.1038/s41467-023-41599-7)
Supplement: Supplementary file 1 — Supplementary Information [file 41467_2023_41599_MOESM1_ESM.pdf]

Supplementary Information

Characterization and structure-based protein engineering of a regiospecific saponin acetyltransferase from *Astragalus membranaceus*

Linlin Wang[1†], Zhihui Jiang[2†], Jiahe Zhang[1†], Kuan Chen[1], Meng Zhang[1], Zilong Wang[1], Binju Wang[2]*, Min Ye[1,3]* and Xue Qiao[1,3]*

[1] State Key Laboratory of Natural and Biomimetic Drugs, School of Pharmaceutical Sciences, Peking University, 38 Xueyuan Road, Beijing 100191, China

[2] State Key Laboratory of Physical Chemistry of Solid Surfaces and Fujian Provincial Key Laboratory of Theoretical and Computational Chemistry, College of Chemistry and Chemical Engineering, Xiamen University, 361005 Xiamen, China

[3] Peking University-Yunnan Baiyao International Medical Research Center, 38 Xueyuan Road, Beijing 100191, China

[†] These authors contributed equally.

*Corresponding authors. E-mail addresses: qiaoxue@bjmu.edu.cn (Xue Qiao), yemin@bjmu.edu.cn (Min Ye), wangbinju2018@xmu.edu.cn (Binju Wang).

Supplementary Methods

Subcellular localization. AmAT7-3 was constructed into pSuper1300-GFP vector using specific primers through homologous recombination and transformed into *Agrobacterium tumefaciens* strain GV2260 (Supplementary Table 2). The strain was cultured in LB medium supplemented with 50 mg/L kanamycin and 50 mg/L rifampin at 28°C until the OD_{600} reached 1.0. The strains were collected and resuspended in MMA buffer (10 mM MES, 10 mM $MgCl_2$, 200 μM acetosyringone, pH 5.6) to incubate in the dark at 28°C for an additional hour. Then, the suspensions were infiltrated into the leaves of 4-week-old tobacco plants. The leaves were cut into small pieces and placed on glass slides, then examined for green fluorescent protein (GFP) expression using a confocal laser scanning microscope (Lecai STELLARIS5; Zeiss, Germany) after 48-72 h. GFP fluorescence was detected with excitation at 488 nm and emission at 500-530 nm.

Optimization of reaction system and kinetic analysis. The catalytic properties of AmAT7-3 and its mutants were investigated using astragaloside IV (**1**) as the acceptor and Ac-CoA as the donor. We optimized the reaction time of AmAT7-3 by analyzing the reaction mixture at various time points between 0-540 min. To determine the optimal reaction buffer, we used different buffers with varying pH ranges: citric acid-sodium citrate buffer (pH 4.0-6.0), Na_2HPO_4-NaH_2PO_4 buffer (pH 6.0-8.0), Tris-HCl buffer (pH 7.0-9.0), and Na_2CO_3-$NaHCO_3$ buffer (pH 9.0-11.0). The reactions were incubated at different temperatures (4-60°C) to study the optimal reaction temperature. To confirm the influence of metal ions, 5 mM of $BaCl_2$, $CoCl_2$, $FeCl_2$, $MnCl_2$, $ZnCl_2$ and EDTA were added into the reaction mixture, respectively. All experiments were conducted in triplicate.

Kinetic studies of AmAT7-3 and its mutants were performed using isoastragaloside II (**1a**) or cyclocephaloside II (**1b**) as acceptors and Ac-CoA as the acetyl donor. The 50-μL reaction mixture were prepared in Na_2HPO_4-NaH_2PO_4 (pH 6.0), containing 2 mM of Ac-CoA, 5-800 μM acyl acceptors, and different concentrations of proteins (0.3 μg AmAT7-3 for **1a**, 0.03 μg AmAT7-3 for **1b**, 0.17 μg AmAT7-3_{A310G} for **1a**, 0.21 μg AmAT7-3_{A310W} for **1b**). The mixture was incubated at 30 °C for 10 min and quenched with 100 μL of ice-cold methanol. All experiments were performed in triplicate. The UHPLC/MS analysis method was same as mentioned above. Michaelis-Menten plots

were fitted to calculate the kinetic parameters. All results were compared to the control and the effects of hydrolysis and acyl migration were deducted.

Chemical analysis. For chemical analysis of Astragalus Root samples, 4.0 g of the powdered material was extracted using 40 mL of methanol by refluxing for 4 hours. The resulting extract was subjected to analysis using a Vanquish Flex UHPLC Dual System coupled with a Charged Aerosol Detector (CAD) (ThermoFisher Scientific, USA). The extracts were passed through a 0.22-μm nylon filter, and 10 μL was subjected to an Agilent Zorbax SB-C18 column (4.6 μm × 250 mm, 5 μm) using 0.1% formic acid (*v*/*v*, A) and acetonitrile (B) as the mobile phase. A gradient program was used: 0-20 min, 20-55% B; 20-25 min, 55-60% B. The flow rate was 1.0 mL/min and the column temperature was 25 °C. The evaporator temperature for CAD was set at 35 °C.

NMR analysis. NMR spectra were recorded on a Bruker AVANCE III-400 instrument at 400 MHz for ^{1}H and 100 MHz for ^{13}C in pyridine-d_5, or a Bruker AVANCE III-600 instrument at 600 MHz for ^{1}H and 150 MHz for ^{13}C in pyridine-d_5. Chemical shifts (δ) are given in parts per million (ppm) and coupling constants (J) are given in hertz (Hz).

Preparation of acetylated products. To synthesize 20(*R*)-ginsenoside Rh1-6'-*O*-acetate (**6a**), compound **6** (198 mg, 1.55 mM) of was dissolved in 2 mL of pyridine with 1.55 mM of acetic anhydride. The mixture was stirred under nitrogen protection at 60 °C for 3 h, followed by quenching with 5 mL of ethyl acetate. The resulting mixture was sequentially washed with 1M HCl (5 mL), saturated sodium bicarbonate (5 mL), and saturated sodium chloride solution (5 mL). The ethyl acetate layer was concentrated and re-dissolved in methanol. The product was pre-purified by thin-layer chromatography using dichloromethane and methanol (6:1) as the mobile phase, and finally purified by HPLC/ELSD using 36% acetonitrile as the eluent. Ziyuglycoside II 6'-*O*-acetate (**3a**) and ziyuglycoside II 4'-*O*-acetate (**3b**) were prepared using the same method except that they were was purified by HPLC/ELSD with 100% ethyl acetate on ReproSil 100 Si column.

To synthesize glycyrrhetinic acid 3-*O*-mono-*β*-D-(6'-acetyl)-glucuronide (**7a**), a scaled-up reaction (30 °C, 1.5 h) was performed in a 500-μL system consisting of Na_2HPO_4-NaH_2PO_4 (pH 6.0), 10 mM DTT, 0.9 mM acetyl coenzyme A, 0.36 mM

glycyrrhetinic acid 3-*O*-mono-*β*-D-glucuronide and 450 mg purified AmAT7-3. The reaction was terminated by adding 1 mL of methanol and centrifuged at 12000 rpm for 20 min to collect the supernatant, which was then concentrated and re-dissolved in 1 mL of methanol. The final product was purified by semi-preparative HPLC on a Zorbax SB-C18 column (4.6×250 mm, 5 μm, Agilent, USA), with ACN (A) and H_2O containing 0.01% trifluoroacetate (B). A linear gradient HPLC elution program was: 0 min, 20% A; 10 min, 65% A; 30 min, 100% A; 40 min, 100% A.

Supplementary Tables

Supplementary Table 1 The template sequences for BLASTn screening.

No.	Name	Species	Accession number	Substrate	Acylation position
1	Ss5MaT1	*Salvia splendens*	AAL50566	Anthocyanin 5-*O*-glucoside	Glc 4'-OH
2	Dm3MAT2	*Dendranthema morifolium*	AAQ63616	Cyanidin 3-*O*-glucoside	Glc 3'/6'-OH
3	TpHCT1A	*Trifolium pratense*	ACI16630	Shikimate, quinate	C5'-OH
4	TpHCT1B	*Trifolium pratense*	ACI28534	Shikimate, quinate	C5'-OH
5	NtHQT	*Nicotiana tabacum*	CAE46932	Quinate, shikimate	C3'/5'-OH
6	CcsHQT	*Cynara cardunculus*	ABK79689	Quinate	C3'-OH
7	SlASAT1	*Solanum lycopersicum*	ALU64003	Sucrose	C-4'-OH
8	SlASAT2	*Solanum lycopersicum*	ALU64014	Acylsucrose	C-3'-OH
9	SlASAT4	*Solanum lycopersicum*	AFM77971.1	Triacylsucrose	C-2'-OH

Supplementary Table 2 Primers for gene cloning and protein expression in *E. coli*, and primers for subcellular localization and transient expression in *N. benthamiana*.

AmAT7-3-F	GGGTCGCGGATCCGAATTCGAGCTCATGAGTTCCTTACGATTAGTTTCAAG
AmAT7-3-R	CGAGTGCGGCCGCAAGCTTGTCGACTCCAATAGAGTGATTGTCCAAGAAC
AmAT8-17-F	GGGTCGCGGATCCGAATTCGAGCTCATGGCTTCTCTGAAAATTCACG
AmAT8-17-R	GAGTGCGGCCGCAAGCTTGTCGACTATGGAAAGGCCTTCACGAAATAG
AmAT7-3_{TEV}-F	GAAAACCTGTATTTTCAGGGCATGAGCAGCCTGCGCCTGGTG
AmAT7-3_{TEV}-R	CGGGCTTTGTTAGCAGCCGGATCTTAGCCAATGCTATGATTGTCTA
AmAT7-3_{GFP}-F	GCAGGGGCCCGGGGTCGACATTTAATGAGCAGCCTGCGCCTGGTGAGC
AmAT7-3_{GFP}-R	CATGGTACCGGATCCACTAGTATTGCCAATGCTATGATTGTCTAAAA
AmAT7-3_{207}-F	GGGGACAAGTTTGTACAAAAAAGCAGGCTTCATGAGCAGCCTGCGCCTGG TGAGC
AmAT7-3_{207}-R	GGGGACCACTTTGTACAAGAAAGCTGGGTTTTAGCCAATGCTATGATTGTC

AmAT7-3-F/R and AmAT8-17-F/R were used for gene cloning from *Astragalus memeranaceus*; AmAT7-3_{TEV}-F/R was used for the construction of the pET28a-TEV-AmAT7-3 recombinant vector; AmAT7-3_{GFP}-F/R was used for the construction of the pSuper1300-GFP-AmAT7-3 recombinant vector; AmAT7-3_{207}-F/R was used for the construction of the pDnor207-AmAT7-3 recombinant vector.

Supplementary Table 3 NMR spectroscopic data (in pyridine d_5, 400 MHz) of compound **3a**.

Position	δ_C (ppm)	δ_H (ppm)
1	39.12, CH_2	2.13, 1.52 m
2	26.95, CH_2	1.46 (m, 2H)
3	89.24, CH	3.33 (dd, J = 11.7, 4.4 Hz, 1H)
4	39.88, C	——
5	56.24, CH	0.86 – 0.80 (m, 1H)
6	19.01, CH_2	1.33, 1.49 m
7	33.88, CH_2	1.60, 1.37
8	40.73, CH	——
9	48.07, CH	1.82 (t, 1H)
10	37.37, C	——
11	24.38, CH_2	2.05 (m, 2H)
12	128.37, CH	5.60 (d, J = 3.7 Hz, 1H)
13	140.34, C	——
14	40.73, C	——
15	29.68, CH_2	1.3 (m, 2H)
16	26.76, CH_2	3.15 (m, 2H)
17	48.67, C	——
18	54.98, CH	3.07 (s, 1H)
19	73.07, C	——
20	42.75, CH	1.59 (m, 1H)
21	27.31, CH_2	1.31 (m, 2H)
22	38.89, CH_2	2.15 (m, 2H)
23	28.57, CH_3	1.24 (s, 3H)
24	17.56, CH_3	1.10 (s, 3H)
25	15.9, CH_3	0.89 (s, 3H)
26	17.23, CH_3	0.94 (s, 3H)
27	25.08, CH_3	1.76 (s, 3H)
28	181.05, C	——
29	27.52, CH_3	1.45 (s, 3H)
30	17.15, CH_3	1.13 (d, 3H)
1'	107.38, CH	4.87 (d, J = 6.5 Hz, 1H)
2'	74.42, CH	4.39 (q, J = 2.9 Hz, 1H)
3'	77.35, CH	5.49 (dd, J = 8.9, 3.4 Hz, 1H)
4'	70.07, CH	4.63 (dd, J = 8.9, 6.5 Hz, 1H)
5'	66.6, CH	4.34 (d, J = 3.7 Hz), 3.89 (d, J = 10.3 Hz, 1H)
1''	171.27, C	——
2''	21.46, CH_3	1.97 (s, 3H)

Supplementary Table 4 NMR spectroscopic data (in pyridine d_5, 400 MHz) of compound **3b**.

Position	δ_C (ppm)	δ_H (ppm)
1	39.13, CH_2	2.12, 1.50 (m, 2H)
2	27.06, CH_2	1.52 (m, 2H)
3	89.41, CH	3.34 (dd, J = 11.8, 4.4 Hz, 1H)
4	39.93, C	——
5	56.28, CH	0.83 (m, 1H)
6	18.98, CH_2	1.32, 1.52
7	33.87, CH_2	1.60, 1.27
8	40.71, CH	——
9	48.06, CH	1.88 (t, 1H)
10	37.34, C	——
11	24.35, CH_2	2.02 (m, 2H)
12	128.36, CH	5.60 (d, J = 3.9 Hz, 1H)
13	140.3, C	——
14	42.46, C	——
15	29.66, CH_2	1.36 (m, 2H)
16	26.74, CH_2	3.15 (m, 2H)
17	48.66, C	——
18	54.96, CH	3.06 (s, 1H)
19	72.82, C	——
20	42.72, CH	1.49 (m, 1H)
21	27.29, CH_2	1.34 (m, 2H)
22	38.87, CH_2	2.15 (m, 2H)
23	28.56, CH_3	1.30 (s, 3H)
24	17.54, CH_3	1.09 (s, 3H)
25	15.86, CH_3	0.86 (s, 3H)
26	17.25, CH_3	0.98 (s, 3H)
27	25.06, CH_3	1.76 (s, 3H)
28	181.04, C	——
29	27.5, CH_3	1.45 (s, 3H)
30	17.13, CH_3	1.13 (d, 3H)
1'	107.95, CH	4.77 (d, J = 7.2 Hz, 1H)
2'	73.05, CH	4.28 (t, J = 2.7 Hz, 1H)
3'	73.52, CH	4.37 (dd, J = 5.9, 3.1 Hz, 1H)
4'	72.86, CH	5.60 (dd, J = 5.9, 3.1 Hz, 1H)
5'	64.7, CH	4.34 (d, J = 3.7 Hz), 3.89 (d, J = 10.3 Hz, 1H)
1"	171.21, C	——
2"	21.46, CH_3	1.98 (s, 3H)

Supplementary Table 5 NMR spectroscopic data (in pyridine d_5, 400 MHz) of compound **6a**.

**Position**	δ_C (ppm)	δ_H (ppm)
1	39.5	1.08/1.74 m
2	28	1.82/1.93 m
3	78.7	3.52 dd (11.6, 4.7)
4	40.4	_
5	61.5	1.39 d (8.2)
6	79.8	4.45 dt (12.1, 5.8)
7	45.6	2.02 d (11.6)/2.54 d (12.1)
8	41.3	_
9	50.3	1.64
10	39.8	_
11	32.3	1.62/2.19 m
12	71	4.02 d (7.6)
13	49.1	2.10 m
14	51.9	
15	31.6	1.26/1.79 m
16	26.7	1.38/1.74 m
17	50.8	2.4
18	17.5	1.01 s
19	17.8	1.09 s
20	73.1	_
21	22.8	1.43 s
22	43.3	1.75 m
23	22.7	2.54 td (14.1, 12.1, 5.5)
24	126.4	5.33 t (7.2)
25	131.2	_
26	25.9	1.71 s
27	17.8	1.65 s
28	31.7	2.08 s
29	16.6	1.57 s
30	17.4	1.31 s
1'	106	5.07 d (7.8)
2'	75.5	4.07 dd (16.1, 8.1)
3'	79.3	4.23 t (8.5)
4'	75.2	4.02 d (7.6)
5'	71.5	3.97 dd (9.8, 4.5)
6'	65.3	5.07 d (7.8)/4.66 dd (11.6, 5.4)
1''	171	
2''	21.1	2.11 s

Supplementary Table 6 NMR spectroscopic data (in pyridine *d*5, 400 MHz) of compound **7a**.

Position	δ_C (ppm)	δ_H (ppm)
1	39.8	3.07 (dt, *J* = 13.6, 3.6 Hz, 2H)
2	26.9	2.29 (dt, *J* = 13.6, 3.5 Hz, 2H)
3	89.4	3.39 (dd, *J* = 11.8, 4.4 Hz, 1H)
4	40.2	_
5	55.6	0.79 (t, 1H)
6	17.9	1.52 (t, *J* = 10.9 Hz, 2H)
7	33.2	1.61 (m, 2H)
8	45.9	_
9	62.4	2.49 (s, 1H)
10	37.6	_
11	199.9	_
12	129	5.98 (s, 1H)
13	170.1	_
14	43.8	_
15	27.2	1.71 (m, 2H)
16	27	0.95 (m, 1H)
17	32.5	_
18	49	2.57- 2.51 (m, 1H)
19	42	2.14 (d, 2H)
20	44.5	_
21	31.9	1.47 (m,2H)
22	38.8	1.71 (m, 2H)
23	28.4	1.29 (s, 3H)
24	17.2	0.99 (s, 3H)
25	17.1	1.24 (s, 3H)
26	19.1	1.08 (s, 3H)
27	23.9	1.45 (s, 3H)
28	29.1	0.79 (s, 3H)
29	29	1.36 (s,3H)
30	179.4	_
1'	107.2	5.02 (d, *J* = 7.7 Hz, 1H)
2'	73.5	4.15 (dd, *J* = 9.5, 7.7 Hz, 1H)
3'	79.13	5.93 (t, *J* = 9.5 Hz, 1H)
4'	71.87	4.60 (t, *J* = 9.7 Hz, 1H)
5'	77.8	4.71-4.67 (m, 1H)
6'	173.2	_
1''	171.1	_
2''	21.5	2.01 (s, 3H)

Supplementary Table 7 Crystallographic data collection and refinement statistics.

	AmAT7-3 (8H8I)	AmAT7-3_{A310G} (8HBT)
**Data collection**		
Space group	R 3 2 :H	R 3 2 :H
Cell dimensions (*a*, *b*, *c*) (Å), (α, β, γ) (°)	(135.63, 135.63, 192.18) (90.00, 90.00, 120.00)	(136.02 136.02 192.12) (90.00, 90.00, 120.00)
Resolution (Å)	74.37-2.03	50.21-1.96
R_{merge} (%)	13.4 (531.7)	11.3 (512.1)
$<I/\delta(I)>$	14.0 (0.7)	16.4 (0.4)
Completeness (%)	99.6 (99.0)	99.8 (99.4)
Multiplicity	19.7 (15.8)	19.0 (12.0)
**Refinement**		
Resolution (Å)	44.77-2.03	50.21-1.96
No. of reflections	2179	2399
R_{work} (%)	21.55	20.88
R_{free} (%)	24.73	22.56
No. atoms		
Protein	3352	3399
Ligand/ion	55/55	55/55
Water	161	181
B-factors		
Protein	47.2	43.6
Ligand/ion	43,47,56,71	41,49,55,63
Rmsd bond lengths (Å)	0.01	0.01
Rmsd bond angles (°)	1.24	1.24
Ramachandran plot residues in favored regions (%)	96.47	96.47

Supplementary Table 8 Total QM/MM energies (a.u.) of the optimized stationary points at the B3LYP-D3/def2-TZVP levels.

Wild-type (a reactive snapshot of conformation-1) (a.u.)	
RC	-2012.7029
TS1	-2012.6813
TS1'-O4'	-2012.6358
IM1	-2012.6856
TS2	-2012.6766
PC	-2012.7038

Wild-type (a reactive snapshot of conformation-2) (a.u.)	
RC	-2127.5003
TS1	-2127.4755
TS1'-O3'	-2127.4531
IM1	-2127.4782
TS2	-2127.4713
PC	-2127.5026

A310W	
RC	-2426.883
TS1	-2426.8483
TS1'-O3'	-2426.8338
IM1	-2426.8695

A310G	
RC	-2177.1777
TS1	-2177.142
TS1'-O4'	-2177.1292
IM1	-2177.1461

Supplementary Figures

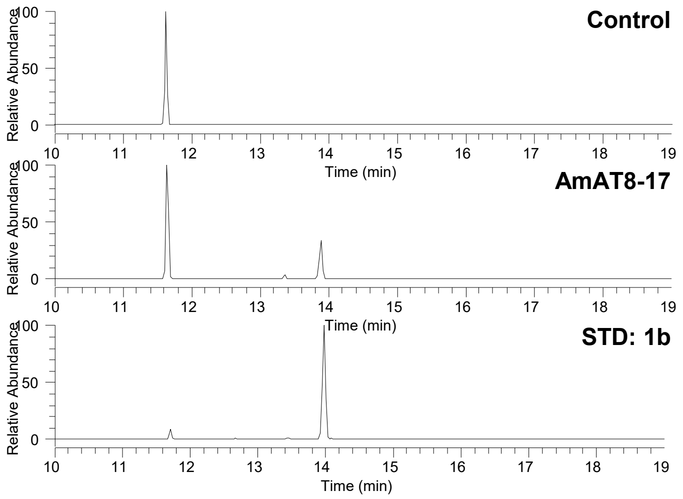

Supplementary Fig. 1 Functional characterization of AmAT8-17.

Astragaloside IV (**1**) was used as acyl acceptor and Ac-CoA as acetyl donor. The function of the recombinant protein was characterized in a mixture including 30 μg protein, 0.1 mM astragaloside IV (**1**), 0.5 mM acetyl-CoA and 0.5 mM dithiothreitol in 50 mM Na_2HPO_4-NaH_2PO_4 buffer (pH 6.0, 100 μL). The reaction was conducted in 30 °C for 30 min. The samples were analyzed following the "UHPLC/MS analysis of enzymatic reaction products" section in Materials and Methods.

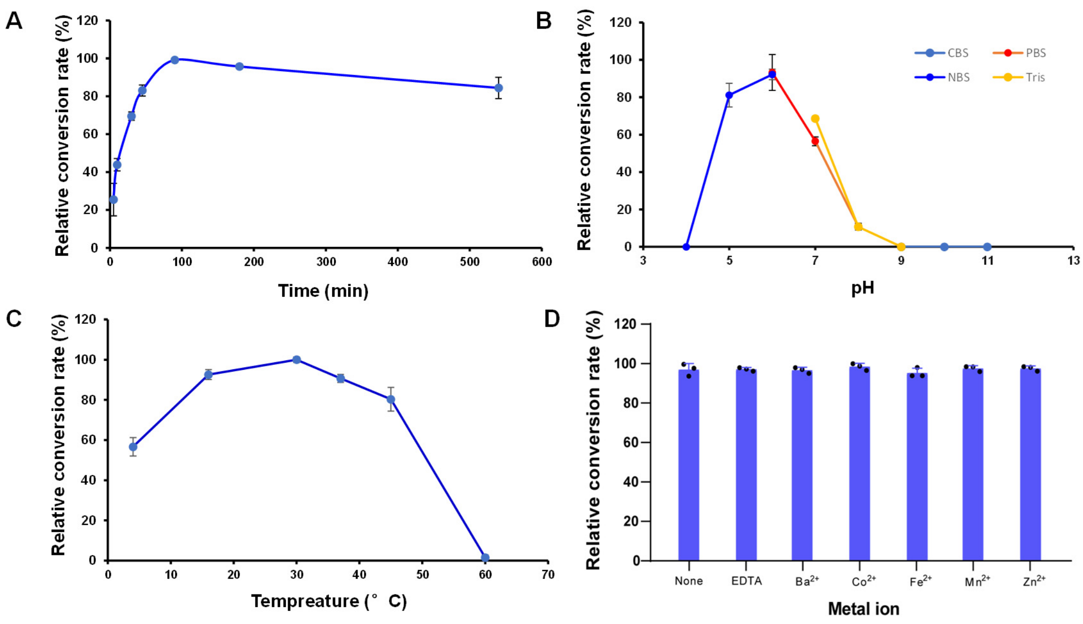

Supplementary Fig. 2 Optimization of the reaction time (A), reaction buffer pH (B), temperature (C) and metal ion (D) for AmAT7-3. Data are presented in mean ± SD (n=2 or 3 independent experiments). Source data are provided as a Source Data file.

Astragaloside IV (**1**) was used as acyl acceptor and Ac-CoA as acetyl donor. The optimized reaction condition was at pH 6.0 (50 mM NaH_2PO_4-Na_2HPO_4) and incubated at 30°C for 30 min. Unless otherwise specified, all parameters remain consistent with the optimal conditions in each panel. NBS: citric acid-sodium citrate buffer (pH 4.0-6.0); PBS: Na_2HPO_4-NaH_2PO_4 buffer (pH 6.0-8.0); Tris: Tris-HCl buffer (pH 7.0-9.0); CBS: Na_2CO_3-$NaHCO_3$ buffer (pH 9.0-11.0).

The relative conversion rate was calculated as follows:

Conversion rate=$A_P/(A_P+A_S)$*100%

Relative conversion rate= C_c/C_m*100%

A_P: peak area of the product; A_S: peak area of the substrate; C_c: conversion rate under the described condition; C_m: the highest conversion rate among all conditions.

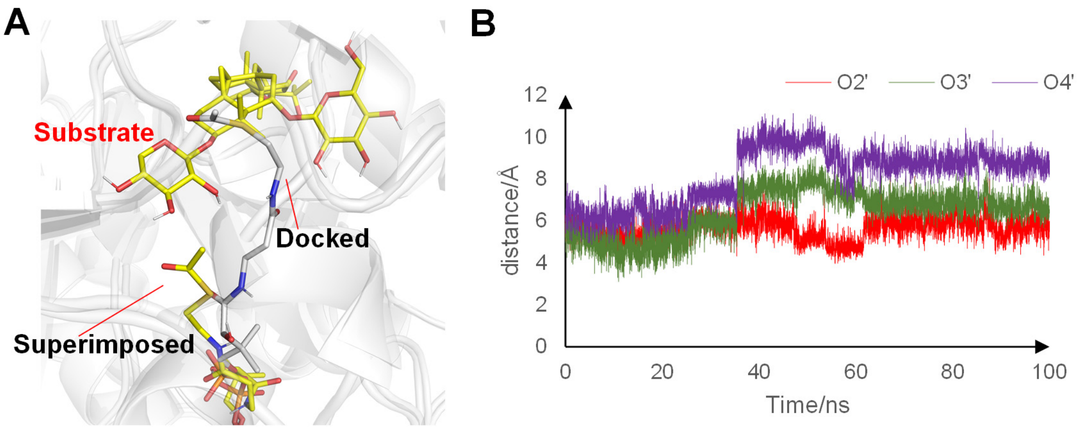

Supplementary Fig. 3 (A) Comparison of the docked (grey) and the superimposed (yellow) structure of Ac-CoA in AmAT7-3. (B) The fluctuation of *C*x-*O*2'/*O*3'/*O*4' distances in 100 ns MD simulation of docked structure. All dockings were performed using the AutoDock Vina tool[2-3].

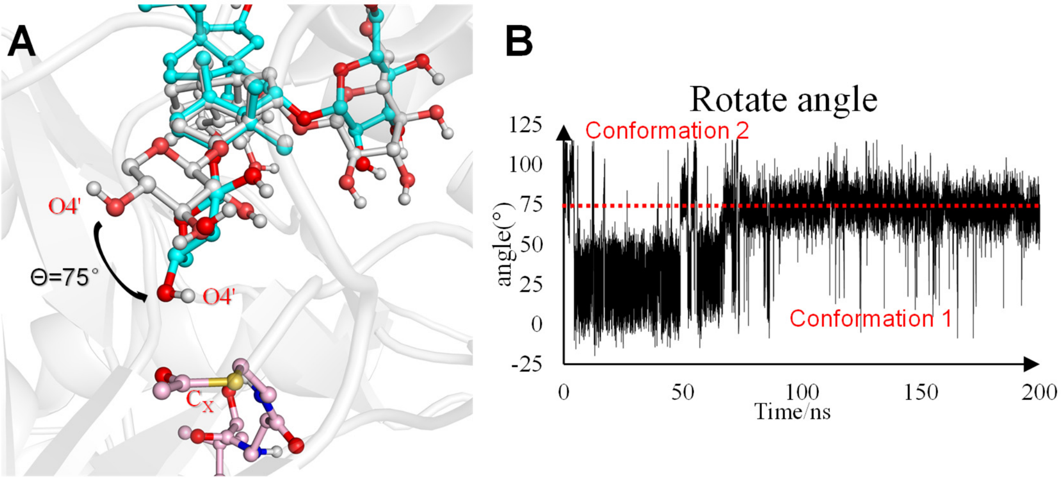

Supplementary Fig. 4 The fluctuation of sugar ring rotation angles for conformation-1 and conformation-2. (A) The complex structure at sugar rotation angle of 75°. The initial crystal structure is showed in grey, and the snapshot from MD simulation is presented in blue. (B) The rotation angle of the xylosyl sugar ring over a 200-ns MD simulation.

In conformation-1, the angle between the sugar ring simulated through MD and the sugar ring in the crystal structure was < 75° (as determined by Multiwfn)[4], in which the *O*3' site of substrate is relatively close to the *C*x of Ac-CoA. In conformation-2, the angle was > 75°, in which the *O*4' site of substrate is relatively close to the *C*x of Ac-CoA.

To evaluate the ratio of conformation-1/conformation-2 and *O*3'-/*O*4'-products, we have further categorized the reactive snapshots based on our QM/MM optimization outcomes and other reports[5]. Concerning *O*3' acetylation, the *C*x-*O*3' distance of less than 3.4 Å is required, alongside a nucleophilic attack angle (formed by the connection between the oxygen on the hydroxyl group of the sugar and the carbonyl group of Ac-CoA) ranging from 90° to 120°. For *O*4' acetylation, the *C*x-*O*4' distance of less than 3.4 Å is necessary, accompanied by a nucleophilic attack angle spanning from 80° to 110°. According to these criteria, we observed a reactive snapshot distribution of 43:4 between conformation-1 and conformation-2 within a 50-ns MD simulation. The ratio of *O*3'-acetylated /*O*4'-acetylated products is approximately 3:1. The computational results are in qualitative agreement with experimental observations, underscoring the prevalence of *O*3' acetylation as the major product.

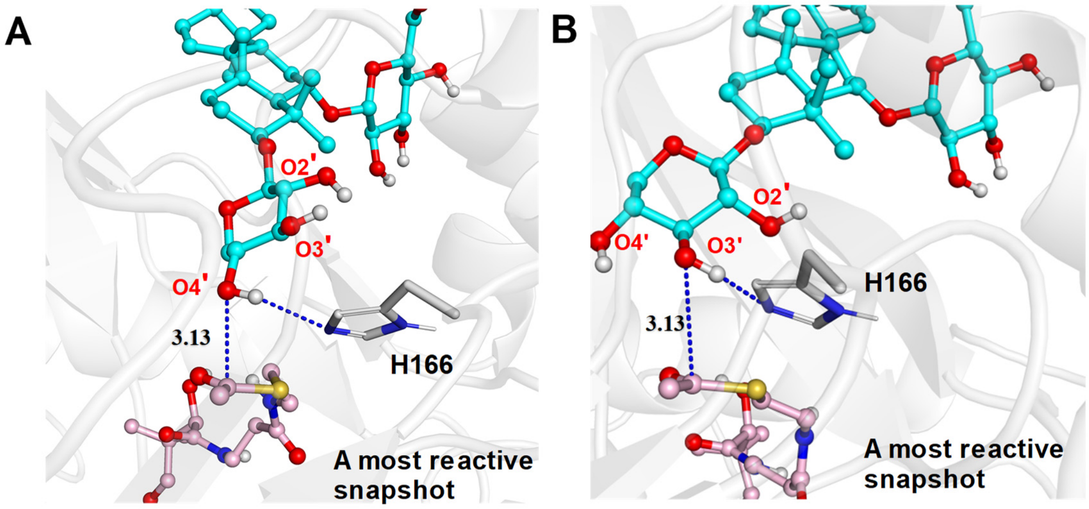

Supplementary Fig. 5 (A) A reactive snapshot in conformation-1 used in QM/MM calculations (the same snapshot as the one in Figure 3G). (B) A reactive snapshot in conformation-2 used in QM/MM calculations (the same snapshot as the one in Figure 3H).

The reactive snapshots feature the a close *C*x-*O*3' or *C*x-*O*4' distance from the near-attack conformation.

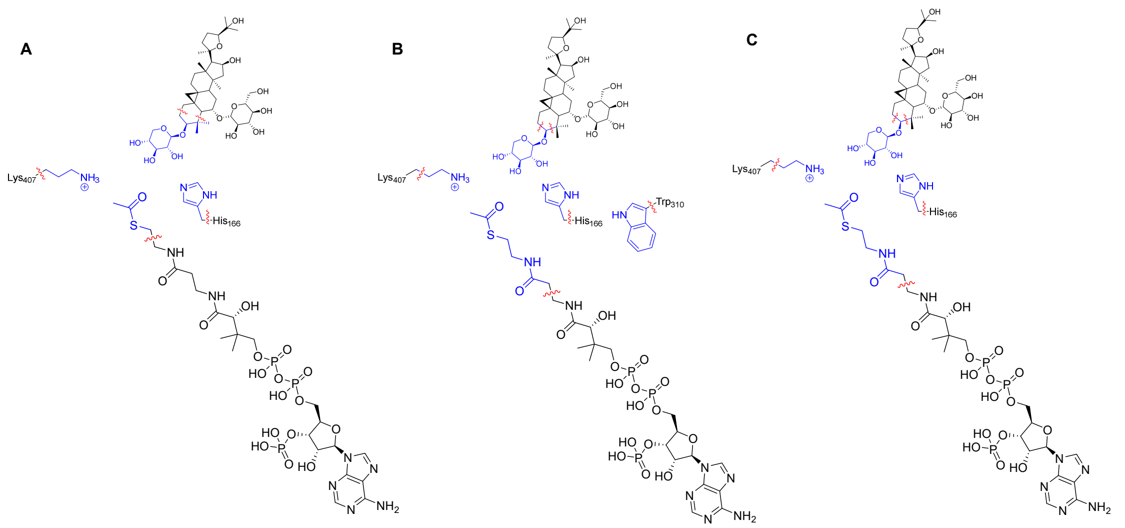

Supplementary Fig. 6 (A) The QM region (in blue) used for AmAT7-3. (B) The QM region (in blue) used for AmAT7-3A310W. (C) The QM region (in blue) used for AmAT7-3A310G. The red line delineates the boundary between the QM region and the MM region.

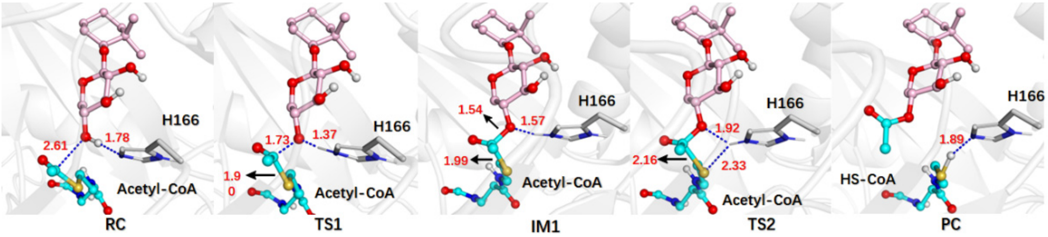

Supplementary Fig. 7 QM/MM-optimized reaction states of the *O*4' acetylation reaction catalyzed by AmAT7-3. RC: reactant complex, TS1: transition state 1, IM1: intermediate 1, TS2: transition state2, PC: product complex.

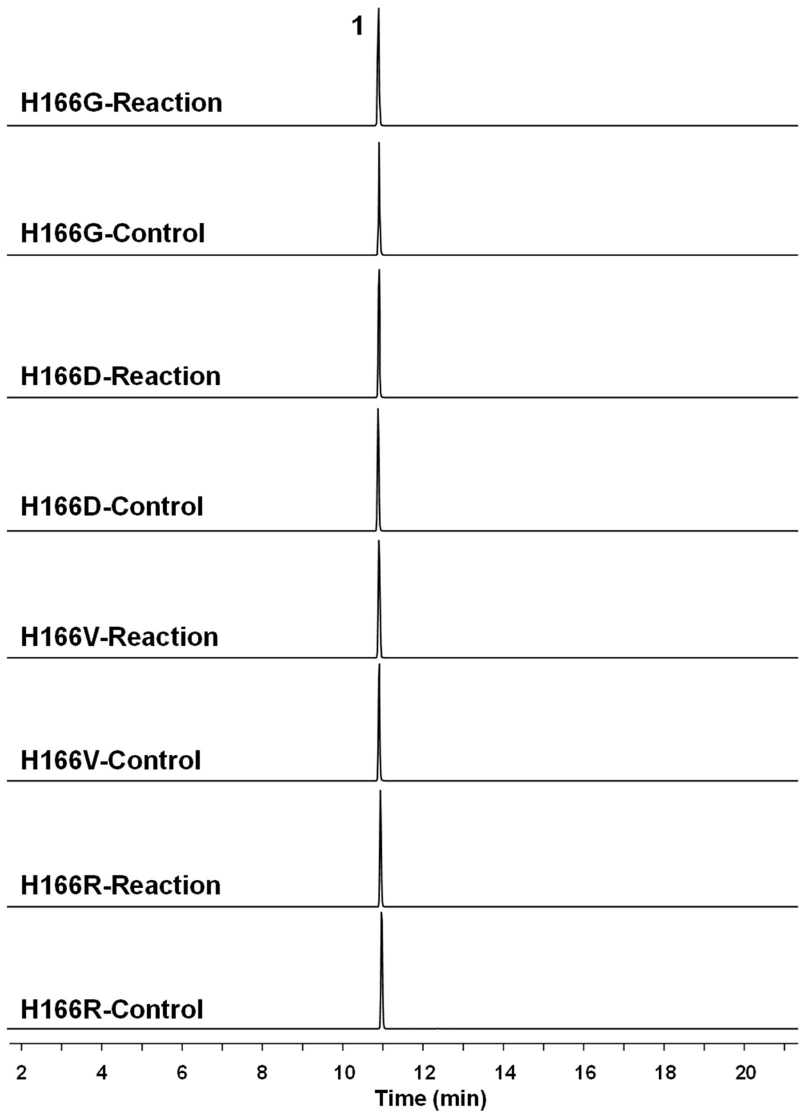

Supplementary Fig. 8 The catalytic activity of H166 mutants.

The reactions were carried out using Astragaloside IV (**1**) as the substrate and Ac-CoA as the acyl donor. The reaction condition was at pH 6.0 (50 mM NaH_2PO_4-Na_2HPO_4), incubated at 30°C for 30 min. Reaction: acetylated reaction catalyzed by AmAT7-3; Control: boiled AmAT7-3 was used in the reaction mixture. LC/MS extracted ion chromatograms were presented by extracting the ions for the substrates and the mono-acetylated products: $[M-H+HCOOH]^-$ (*m/z* 829.45), $[M+Ac-H+HCOOH]^-$ (*m/z* 871.46).

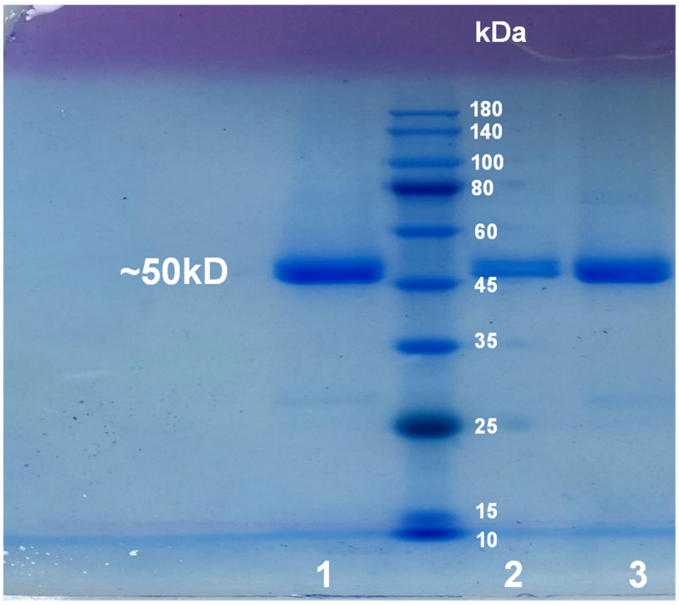

Supplementary Fig. 9 SDS-PAGE of AmAT7-3, AmAT7-3$_{A310G}$, and AmAT7-3$_{A310W}$ proteins.

Sample 1-3 was AmAT7-3, AmAT7-3$_{A310G}$ and AmAT7-3$_{A310W}$ purified by size exclusion chromatography, respectively.

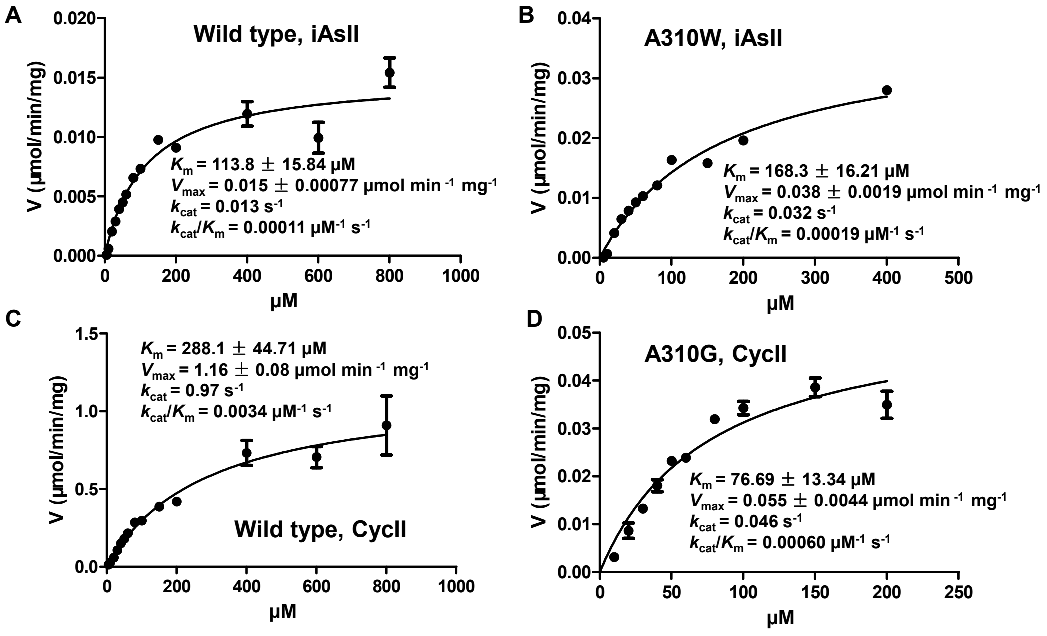

Supplementary Fig. 10 Kinetic analysis of AmAT7-3, AmAT7-3$_{A310W}$ and AmAT7-3$_{A310G}$. The apparent K_m value was determined using isoastragaloside II (**1a**, iAsII) or cyclocephaloside II (**1b**, CycII) as the acyl acceptor and Ac-CoA as the acyl donor at 30 °C for 10 min in Na_2HPO_4-NaH_2PO_4 (pH 6.0). The samples were analyzed following the "UHPLC/MS analysis of enzymatic reaction products" section in Materials and Methods.

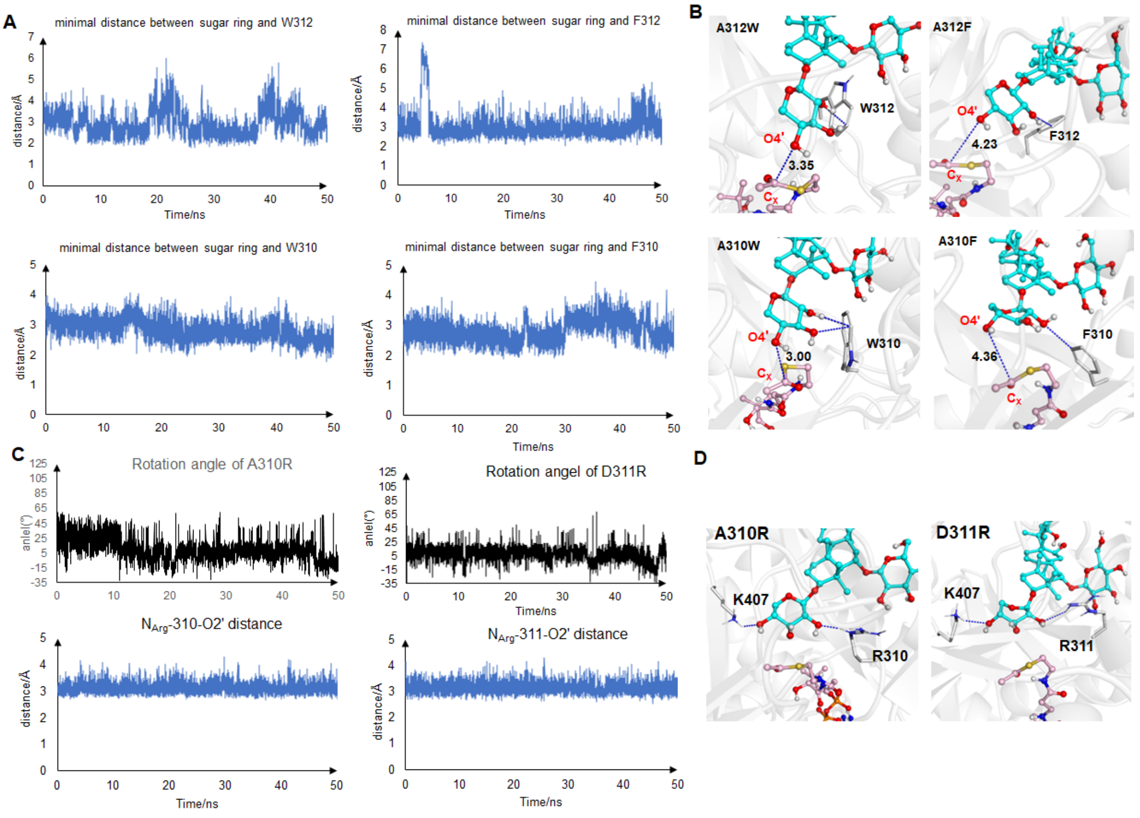

Supplementary Fig. 11 (A) The fluctuation of minimal distance between the hydroxyl group of the sugar and key residues in A310W/F and A312W/F mutants. (B) Representative snapshots of the reactant complexes for A310W/F and A312W/F mutants. (C) The fluctuation of sugar rotation angle and minimal distance between the *O*2' of the sugar and *N* atom of R310/R311 in A310R/D311R mutants. (D) Representative snapshots of the reactant complexes for A310R/D311R mutants.

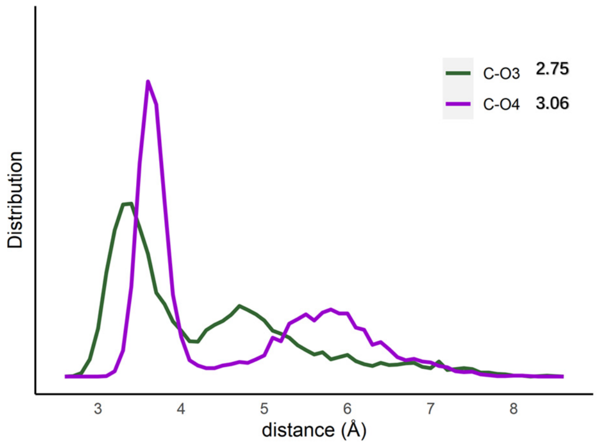

Supplementary Fig. 12 The distance population between acyl *C* of Ac-CoA and sugar *O* sites of astragaloside IV in A310G.

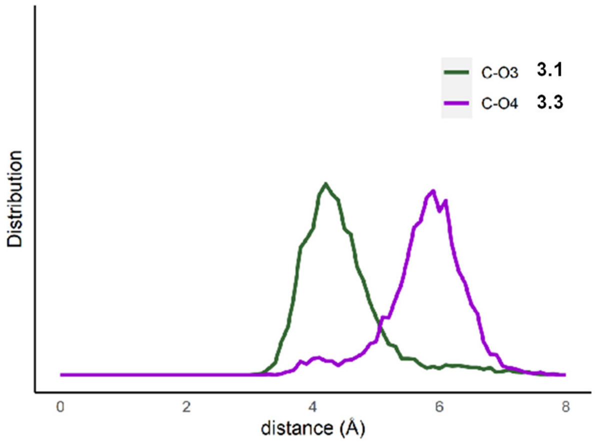

Supplementary Fig. 13 The distance population between acyl *C* of Ac-CoA and sugar *O* sites of astragaloside IV in A310W.

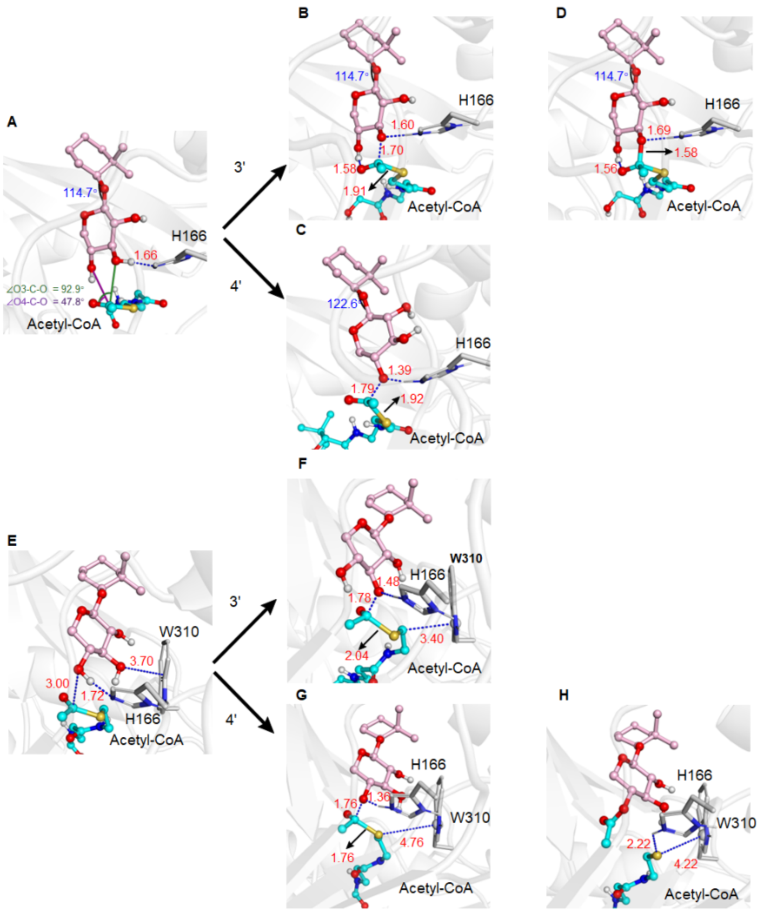

Supplementary Fig. 14 Site selectivity of A310G and A310W.

(A) The reactant complex of A310G. (B) Transition state 1 of C3' acetylation of astragaloside IV in A310G. (C) Transition state 1 of C4' acetylation of astragaloside IV in A310G. (D) Intermediate of C3' acetylation of astragaloside IV in A310G. (E) The reactant complex of A310W. (F) Transition state 1 of C3' acetylation of astragaloside IV in A310W. (G) Transition state 1 of C4' acetylation of astragaloside IV in A310W. (H) Intermediate of C4' acetylation of astragaloside IV in A310W.

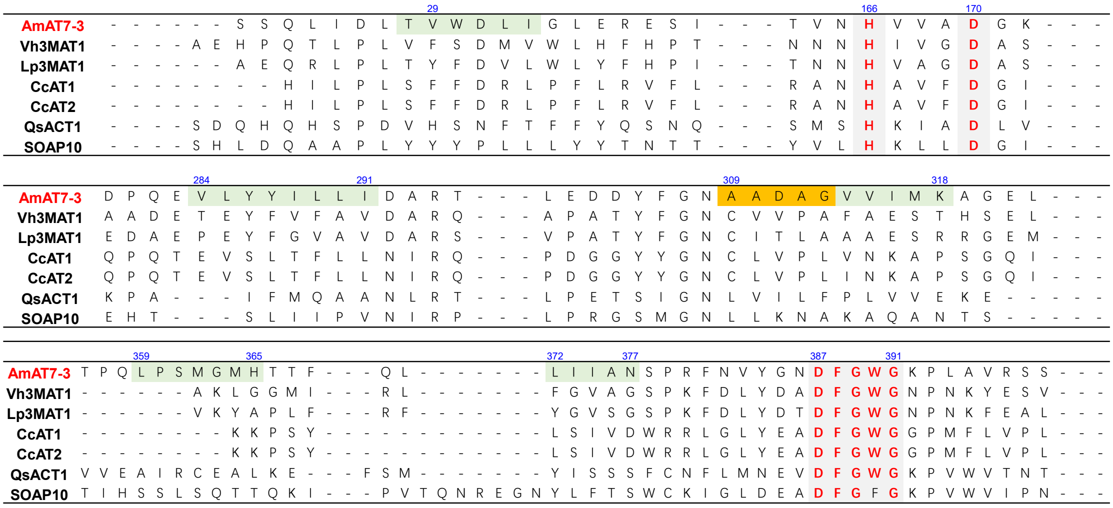

Supplementary Fig. 15 Sequence analysis for AmAT7-3 and other flavonoid and saponin acetyltransferases.

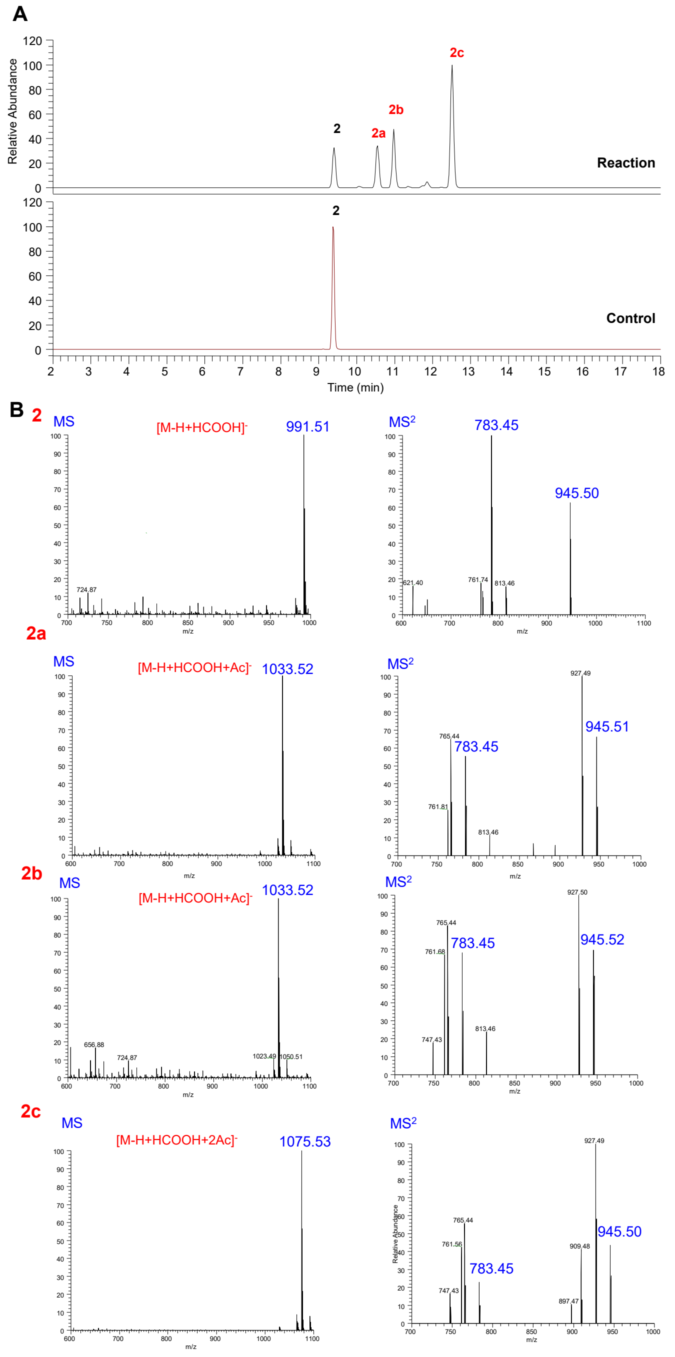

Supplementary Fig. 16 UPLC/MS chromatograms of the reaction mixtures for AmAT7-3 using **2** as the acyl acceptor, and the MS and MS/MS spectra of product **2a**/**2b**/**2c**.

Reaction, Ac-CoA was added as the acyl donor; control, the acyl donor is absent. MS and MS/MS spectra of **2a**/**2b**/**2c** were obtained in negative ion mode.

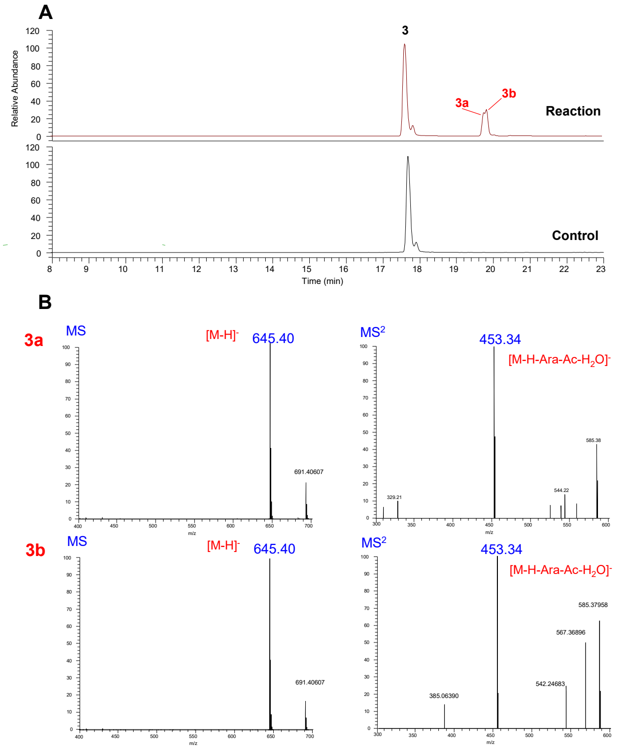

Supplementary Fig. 17 UPLC/MS chromatograms of the reaction mixtures for AmAT7-3 using **3** as the acyl acceptor, and the MS and MS/MS spectra of product **3a** and **3b**.

Reaction, Ac-CoA was added as the acyl donor; control, the acyl donor is absent. MS and MS/MS spectra of **3a** and **3b** were obtained in negative ion mode.

The samples were separated on a Waters T3 column (2.1×100 mm, 1.8 µm) using 0.1% formic acid (*v*/*v*, A) and acetonitrile (B) at 50°C. A gradient program was used: 0-1 min, 10% B; 1-19 min, 10%-60% B; 19-20 min, 60%-100% B; 20-22 min, 100% B. The flow rate was 0.3 mL/min. MS analysis was performed in the same method following the "UHPLC/MS analysis of enzymatic reaction products" section in Materials and Methods.

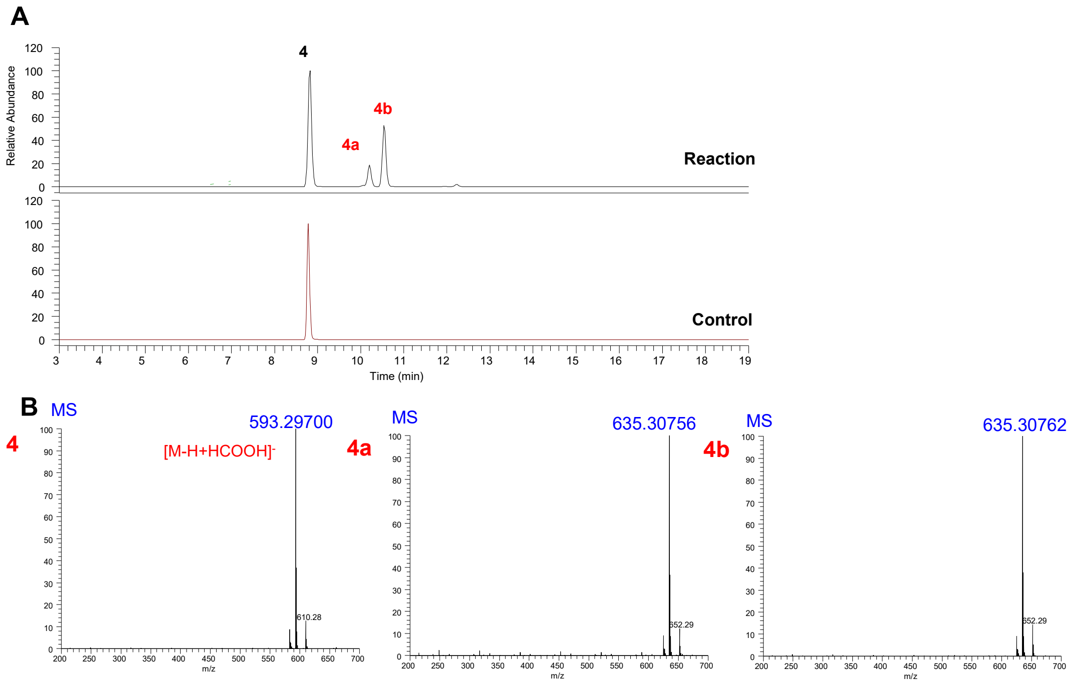

Supplementary Fig. 18 UPLC/MS chromatograms of the reaction mixtures for AmAT7-3 using **4** as the acyl acceptor, and the MS spectra of product **4a** and **4b**. Reaction, Ac-CoA was added as the acyl donor; control, the acyl donor is absent. MS spectra of **4a** and **4b** were obtained in negative ion mode. Due to the difficulties in obtaining MS/MS data, the acetylated products **4a**/**4b** were identified based on accurate molecular weights (Calcd. 635.30730, Δ 0.5 ppm).

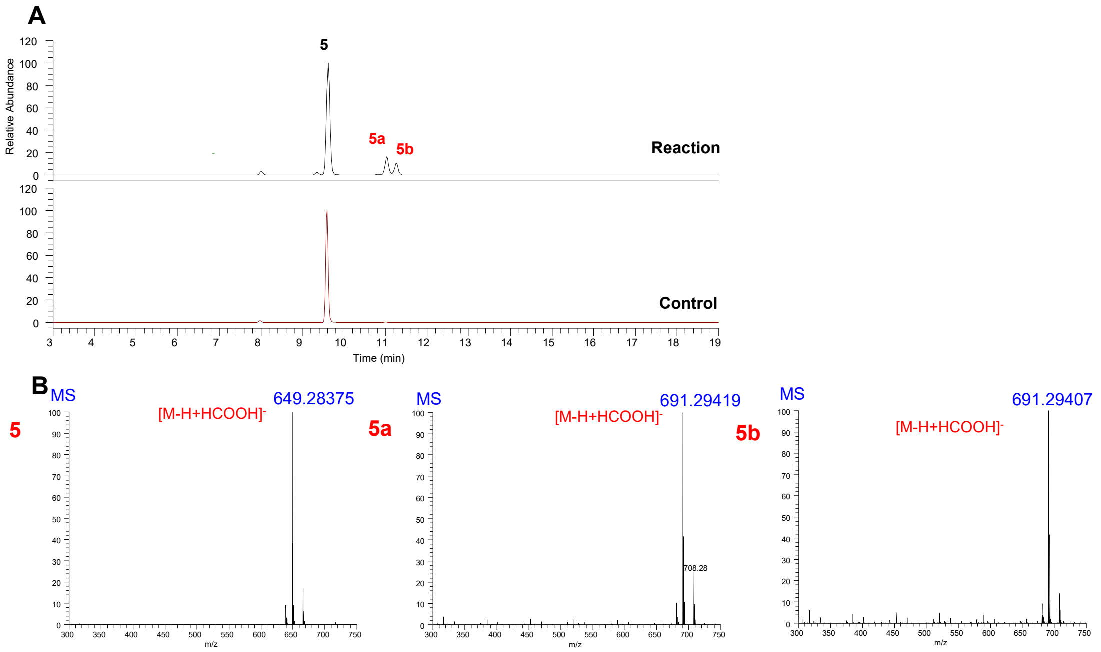

Supplementary Fig. 19 UPLC/MS chromatograms of the reaction mixtures for AmAT7-3 using **5** as the acyl acceptor, and the MS spectra of product **5a**/**5b**. Reaction, Ac-CoA was added as the acyl donor; control, the acyl donor is absent. MS spectra of **5a**/**5b** were obtained in negative ion mode. Due to the difficulties in obtaining MS/MS data, the acetylated products **5a**/**5b** were identified based on accurate molecular weights (Calcd. 691.2971, Δ 4.0 ppm).

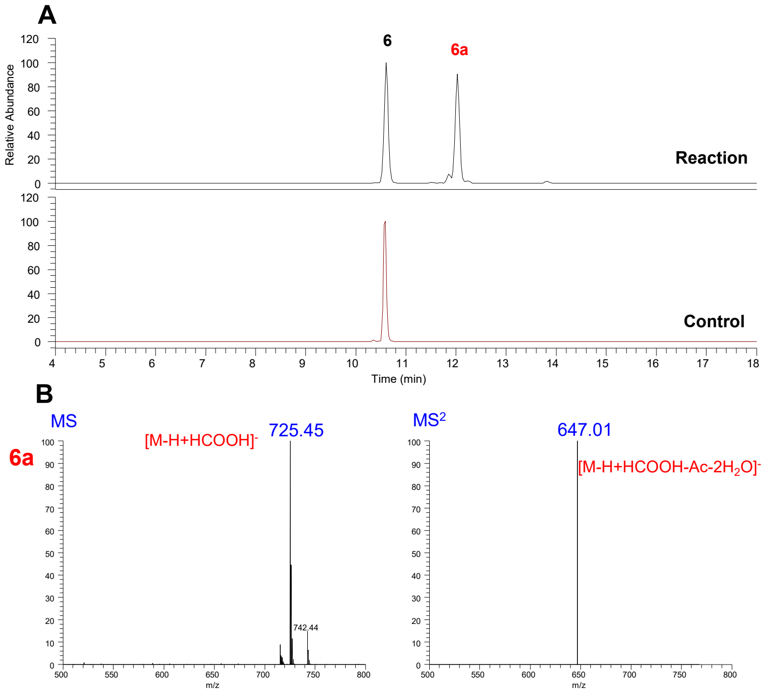

Supplementary Fig. 20 UPLC/MS chromatograms of the reaction mixtures for AmAT7-3 using **6** as the acyl acceptor, and the MS and MS/MS spectra of product **6a**. Reaction, Ac-CoA was added as the acyl donor; control, the acyl donor is absent. MS and MS/MS spectra of **6a** were obtained in negative ion mode.

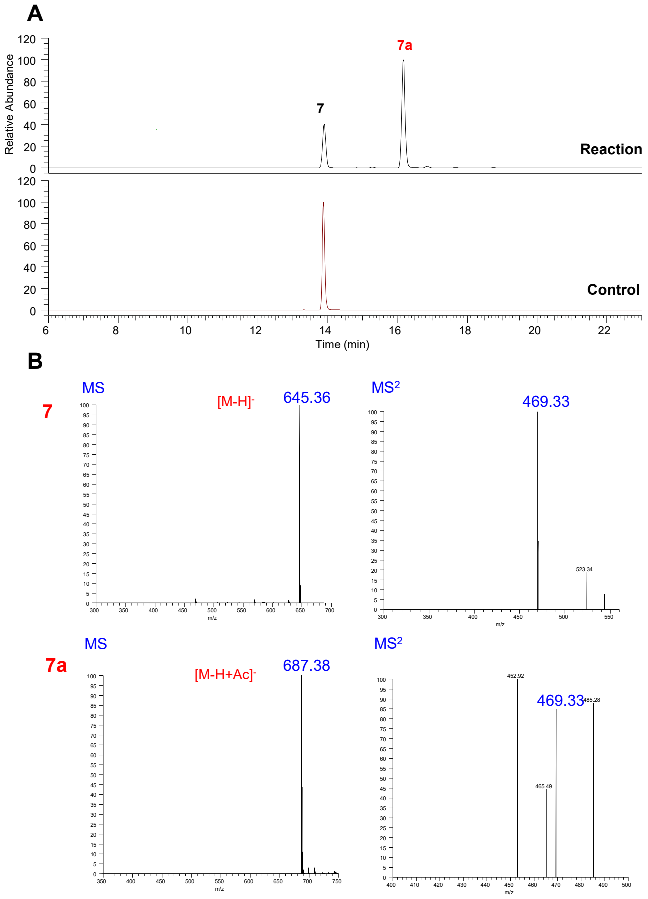

Supplementary Fig. 21 UPLC/MS chromatograms of the reaction mixtures for AmAT7-3 using **7** as the acyl acceptor, and the MS and MS/MS spectra of product **7a**. Reaction, Ac-CoA was added as the acyl donor; control, the acyl donor is absent. MS and MS/MS spectra of **7a** were obtained in negative ion mode.

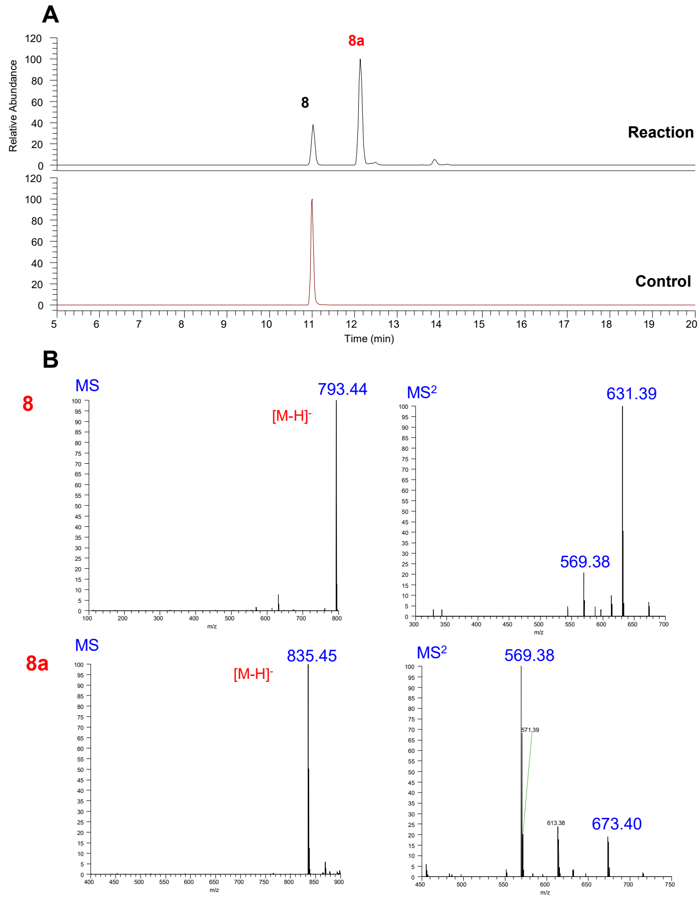

Supplementary Fig. 22 UPLC/MS chromatograms of the reaction mixtures for AmAT7-3 using **8** as the acyl acceptor, and the MS and MS/MS spectra of product **8a**. Reaction, Ac-CoA was added as the acyl donor; control, the acyl donor is absent. MS and MS/MS spectra of **8a** were obtained in negative ion mode.

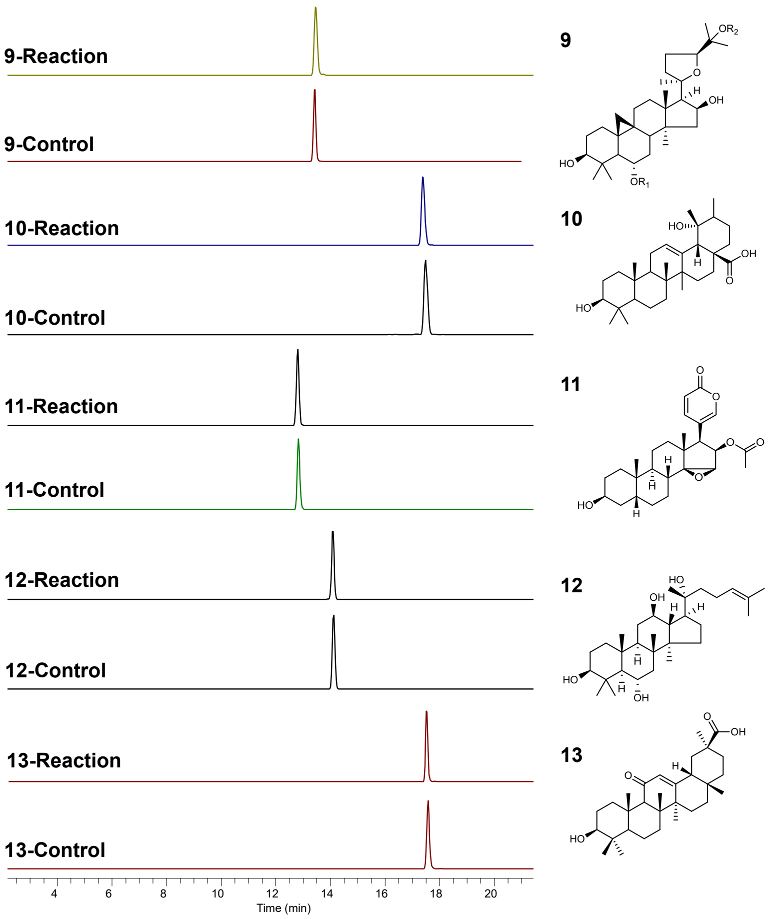

Supplementary Fig. 23 Reaction and chemical structures of compound 9-13 catalyzed by AmAT7-3.

Reference standards cycloastragenol (**9**), pomolic acid (**10**), cinobufotalin (**11**), 20(*R*)-protopanaxatriol (**12**) and 18β-glycyrrhetinic acid (**13**) were purchased from Must Bio-Technology (Chengdu, China). LC/MS extracted ion chromatograms were presented by extracting the ions for the substrates and the products: $[M+Na]^+$ (*m/z* 513.35), $[M+Ac+Na]^+$ (*m/z* 555.36) for **9**; $[M-H]^-$ (*m/z* 471.35), $[M+Ac\ -H]^-$ (*m/z* 513.36) for **10**; $[M+H]^+$ (*m/z* 443.24), $[M+Ac+H]^+$ (*m/z* 485.25) for **11**; $[M-H+HCOOH]^-$ (*m/z* 521.38), $[M+Ac-H+HCOOH]^-$ (*m/z* 563.39) for **12**; and $[M-H]^-$ (*m/z* 469.33), $[M+Ac-H]^-$ (*m/z* 511.34) for **13**.

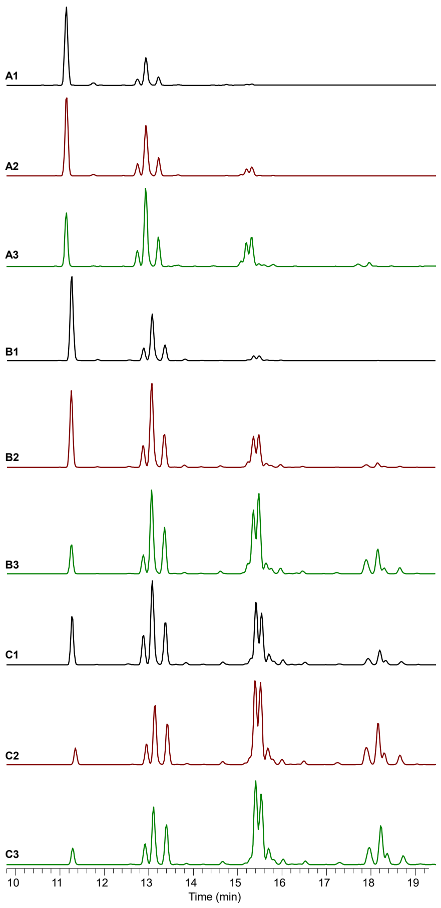

Supplementary Fig. 24 Optimization of chemical acetylation conditions for compound **6**.

The reaction was carried out in 600 μL pyridine with 3.98 mg **6** and 72 mM of acetic anhydride. The mixture was stirred under nitrogen protection at (A1) 4 °C, 0.5 h, (A2) 4 °C, 2 h, (A3) 4 °C, 6 h, (B1) 30 °C, 0.5 h, (B2) 30 °C, 2 h, (B3) 30 °C, 6 h, (C1) 70 °C, 0.5 h, (C2) 70 °C, 2 h, (C3) 70 °C, 6 h.

LC/MS extracted ion chromatograms were presented by extracting the ions for the substrates and the mono-, di- and tri- acetylated products: $[M\text{-}H\text{+}HCOOH]^-$ (*m*/*z* 683.44), $[M\text{+}Ac\text{-}H\text{+}HCOOH]^-$ (*m*/*z* 725.45), $[M\text{+}2Ac\text{-}H\text{+}HCOOH]^-$ (*m*/*z* 767.46), and $[M\text{+}3Ac\text{-}H\text{+}HCOOH]^-$ (809.47).

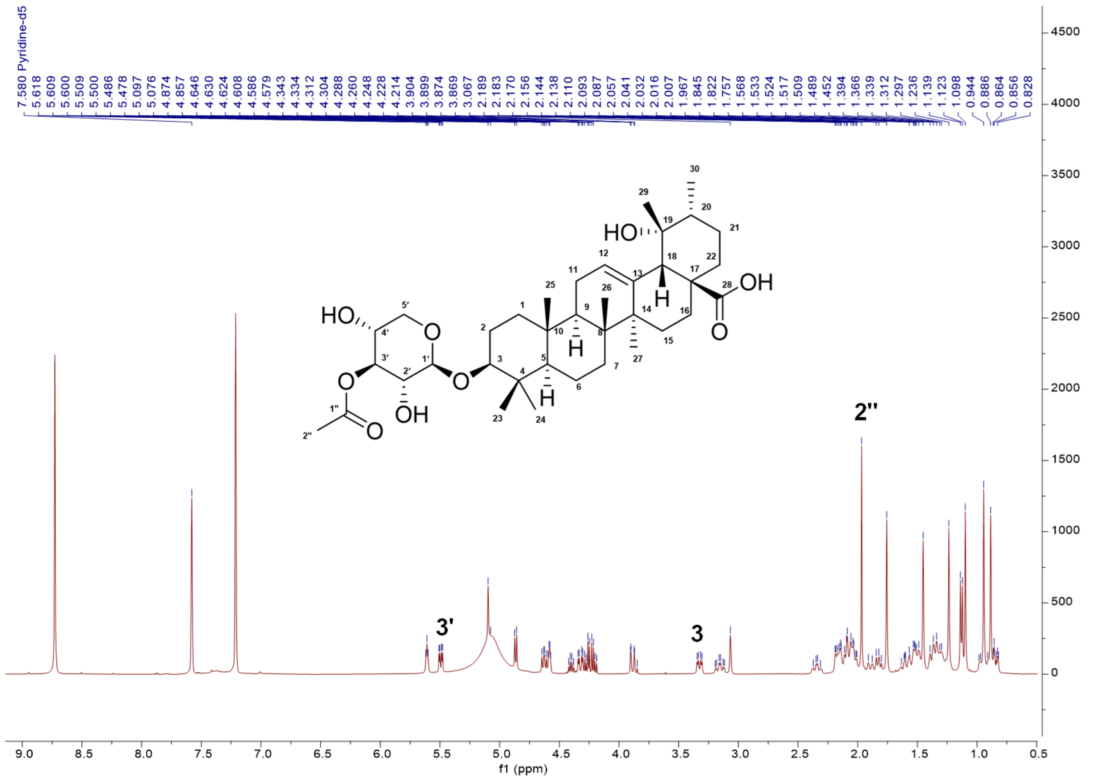

Supplementary Fig. 25 ^{1}H NMR spectrum of **3a** in pyridine-d_5 (600 MHz).

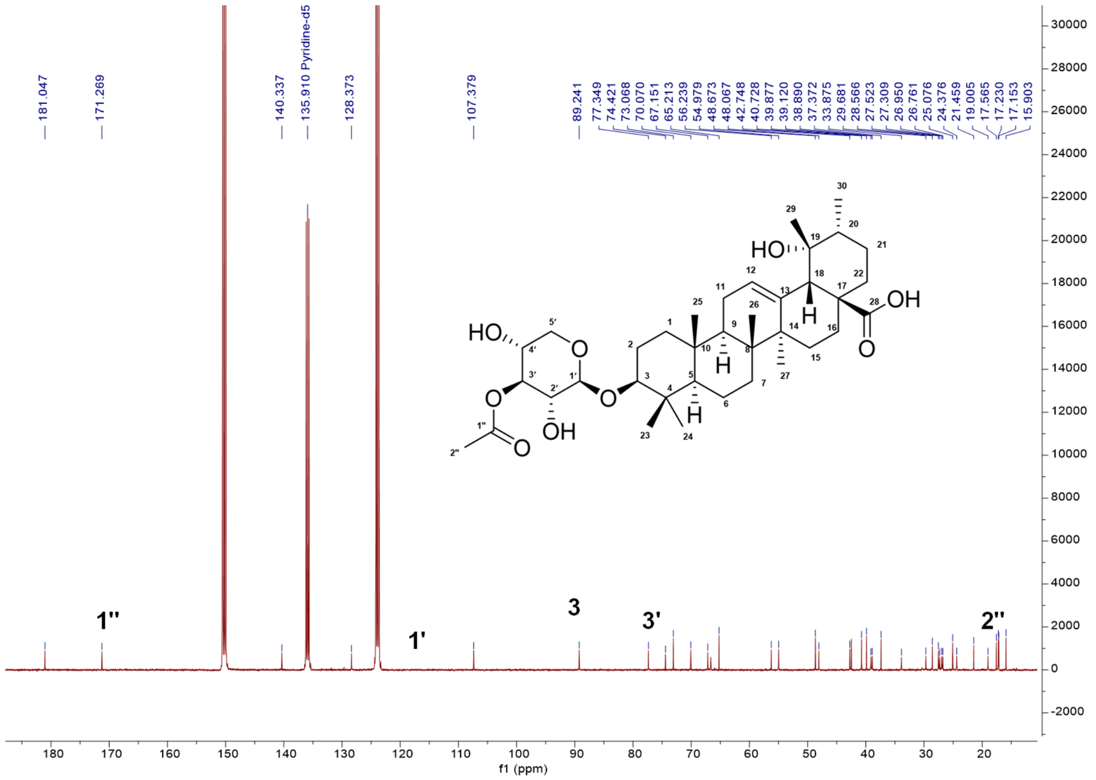

Supplementary Fig. 26 ^{13}C NMR spectrum of **3a** in pyridine-d_5 (150 MHz).

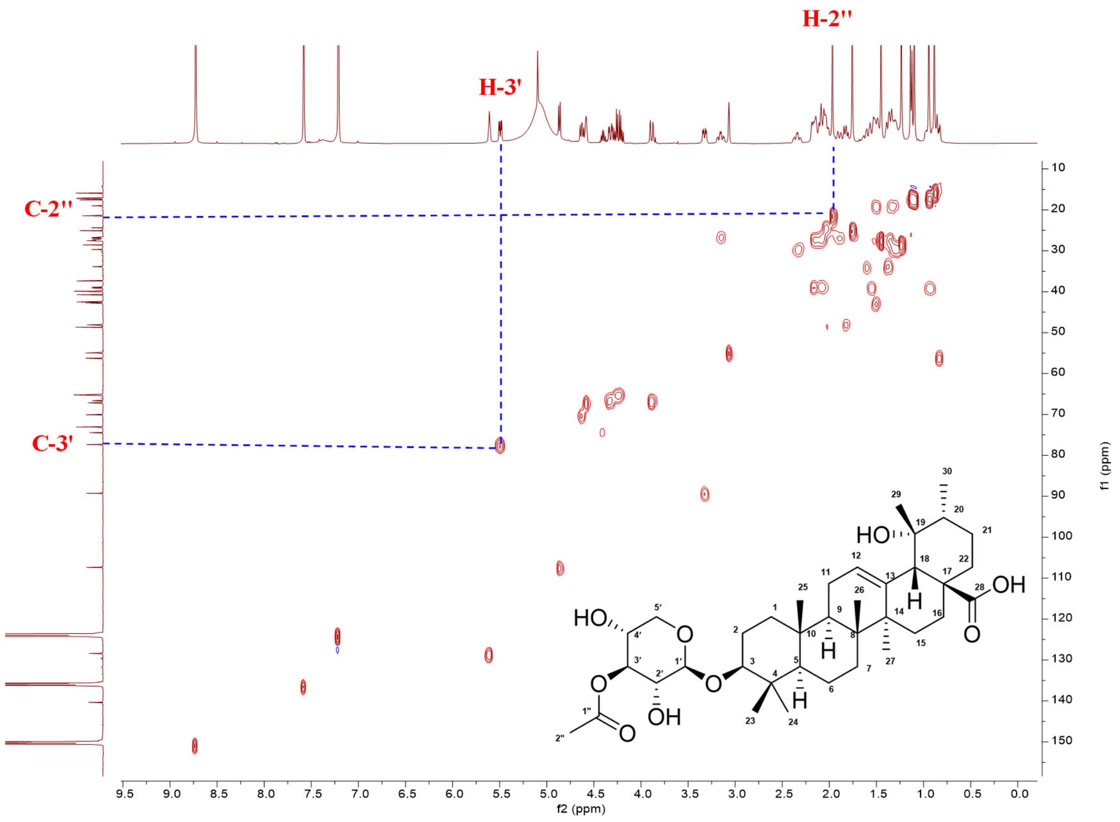

Supplementary Fig. 27 HSQC spectrum of **3a** in pyridine-d_5 (600 MHz).

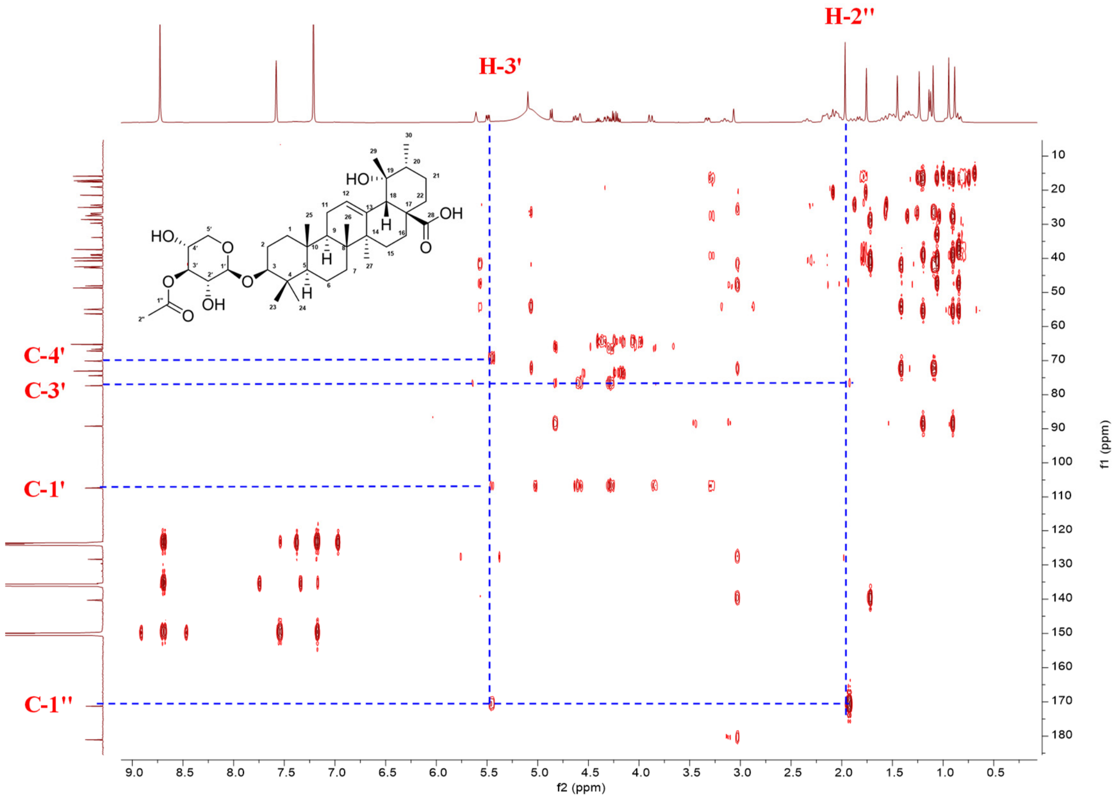

Supplementary Fig. 28 HMBC spectrum of **3a** in pyridine-d_5 (600 MHz).

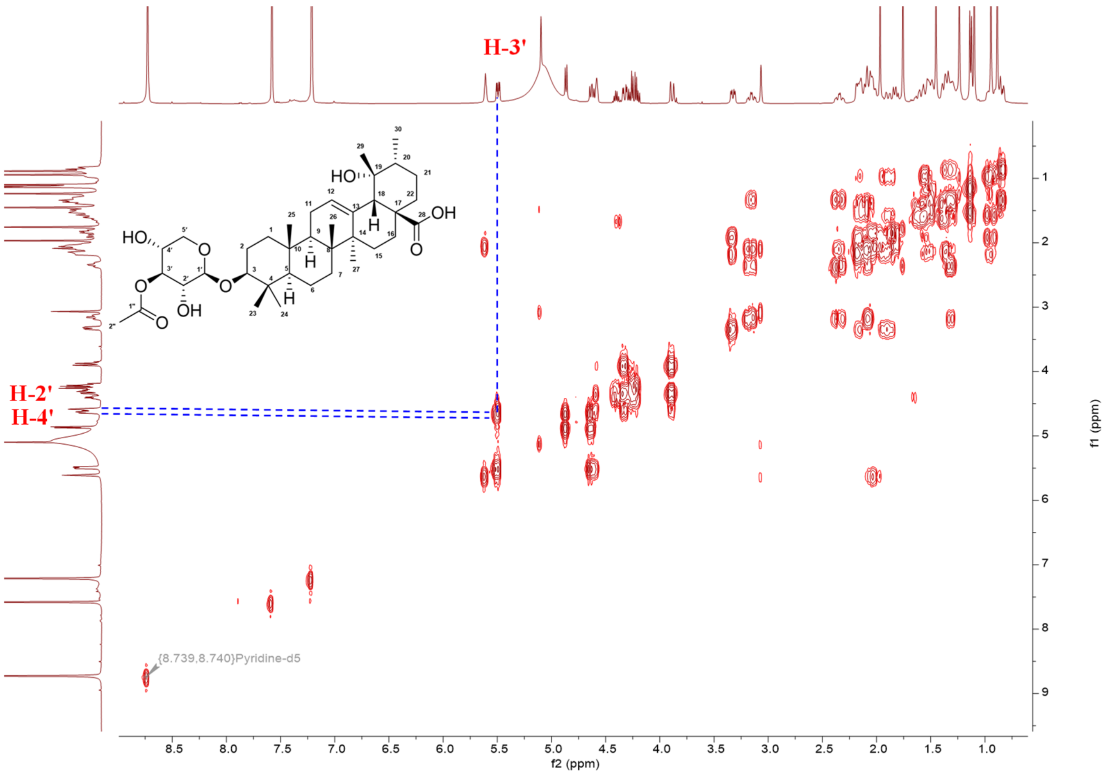

Supplementary Fig. 29 ^{1}H-^{1}H COSY spectrum of **3a** in pyridine-d_5 (400 MHz).

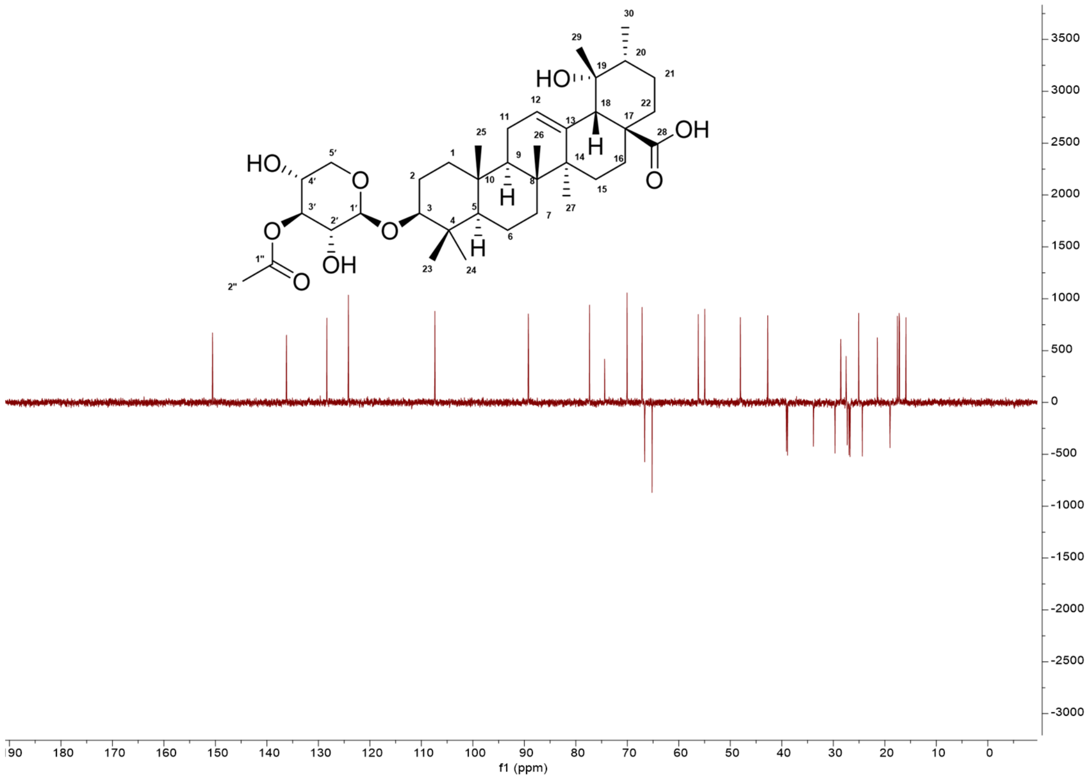

Supplementary Fig. 30 DEPT 135 spectrum of **3a** in pyridine-d_5 (400 MHz).

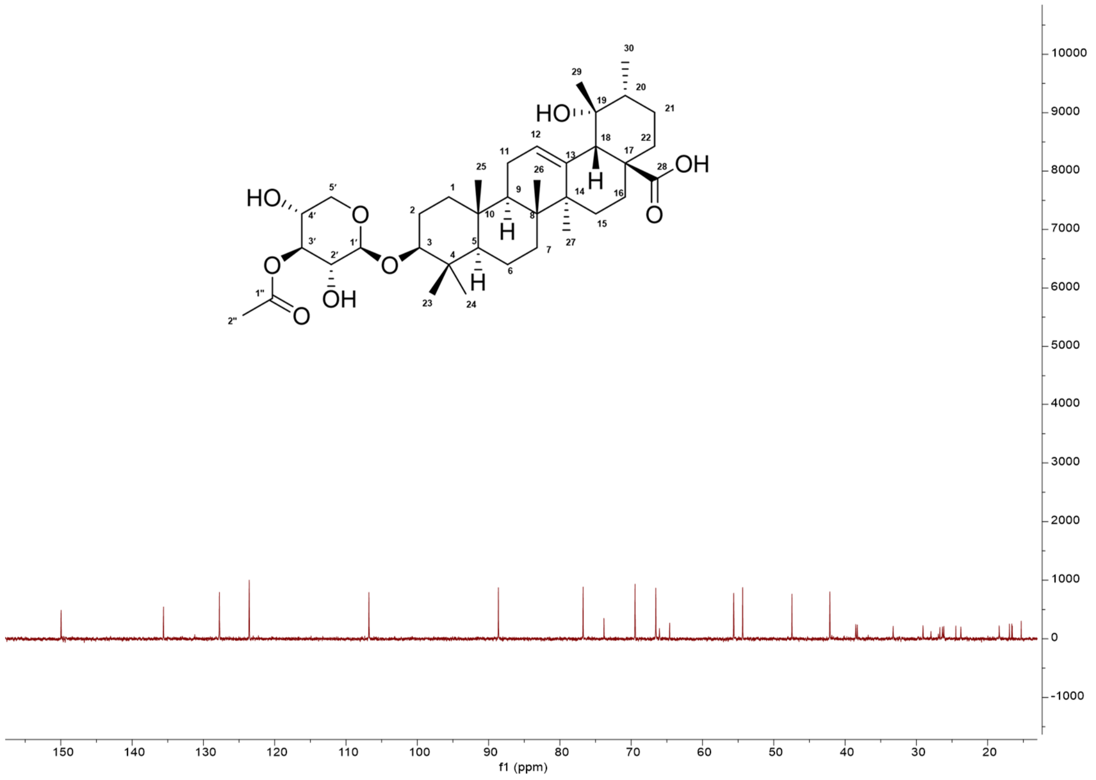

Supplementary Fig. 31 DEPT 90 spectrum of **3a** in pyridine-d_5 (400 MHz).

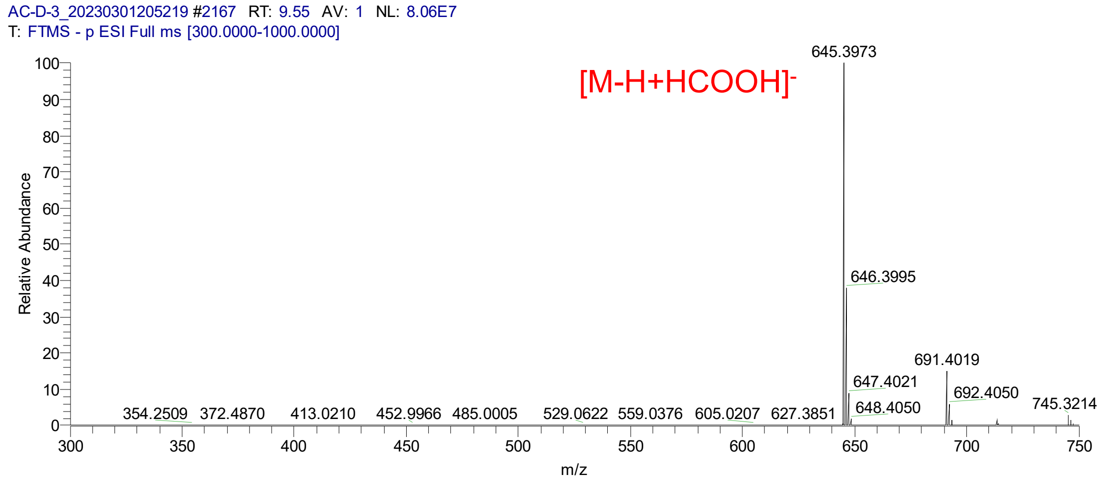

Supplementary Fig. 32 (-)ESI-HR-MS spectrum of **3a**.

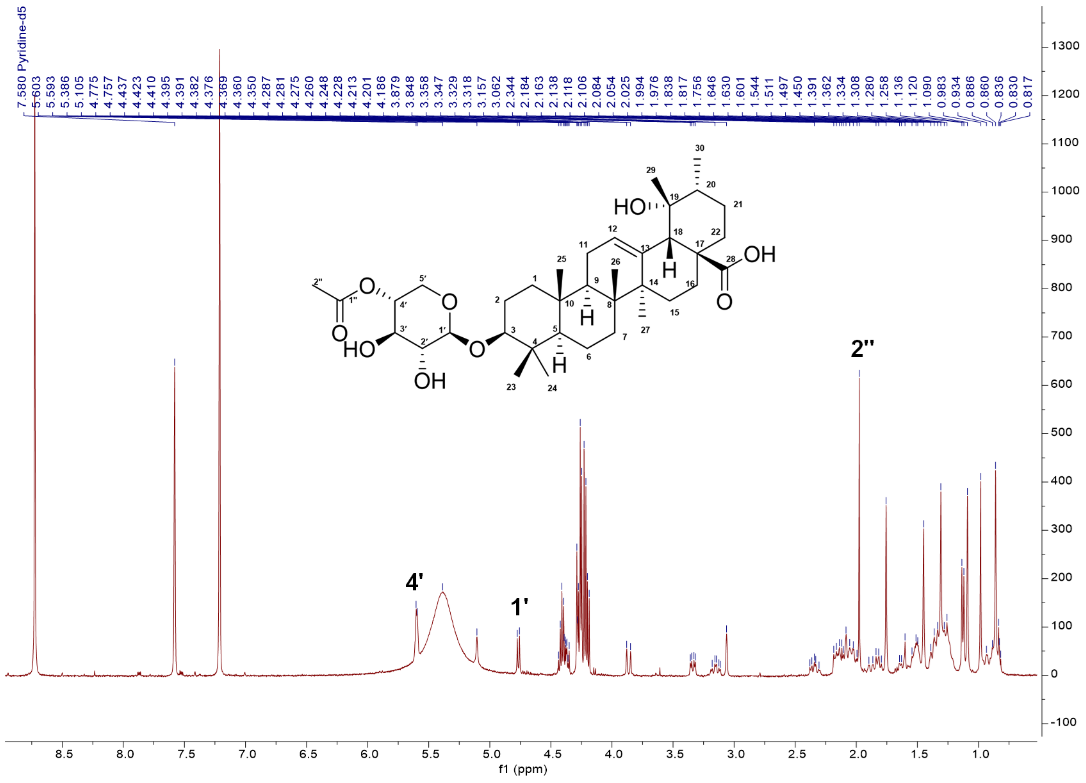

Supplementary Fig. 33 ^{1}H NMR spectrum of **3b** in pyridine-d_5 (400 MHz).

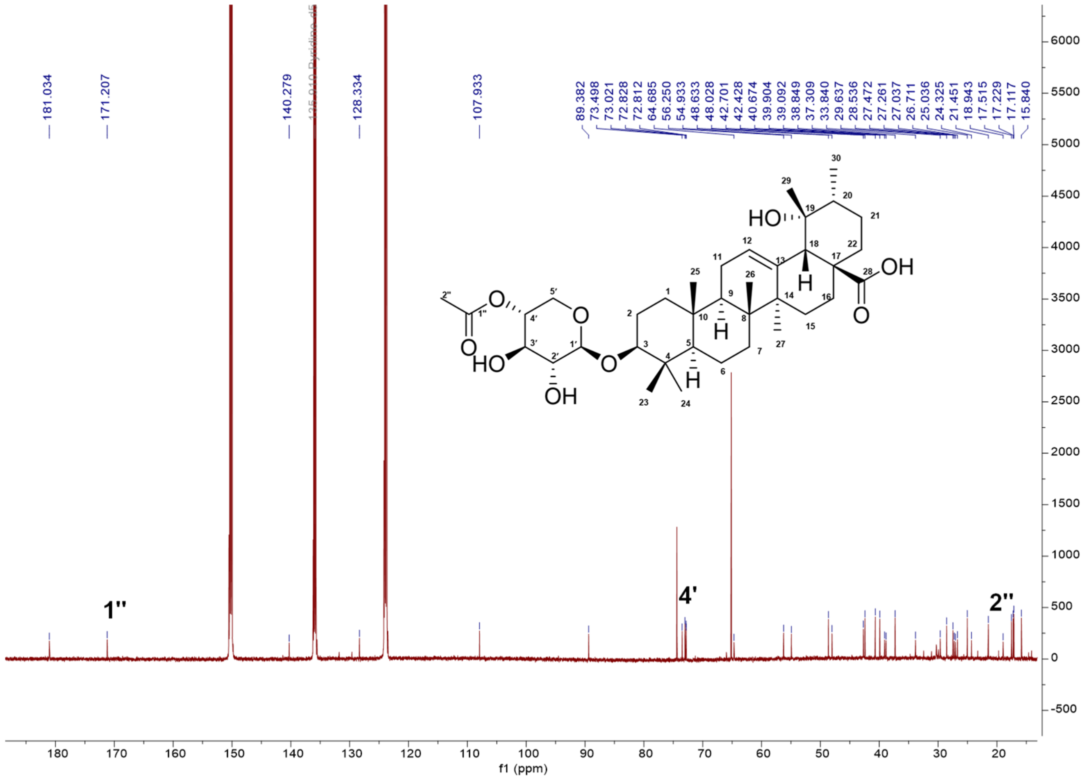

Supplementary Fig. 34 ^{13}C NMR spectrum of **3b** in pyridine-d_5 (100 MHz).

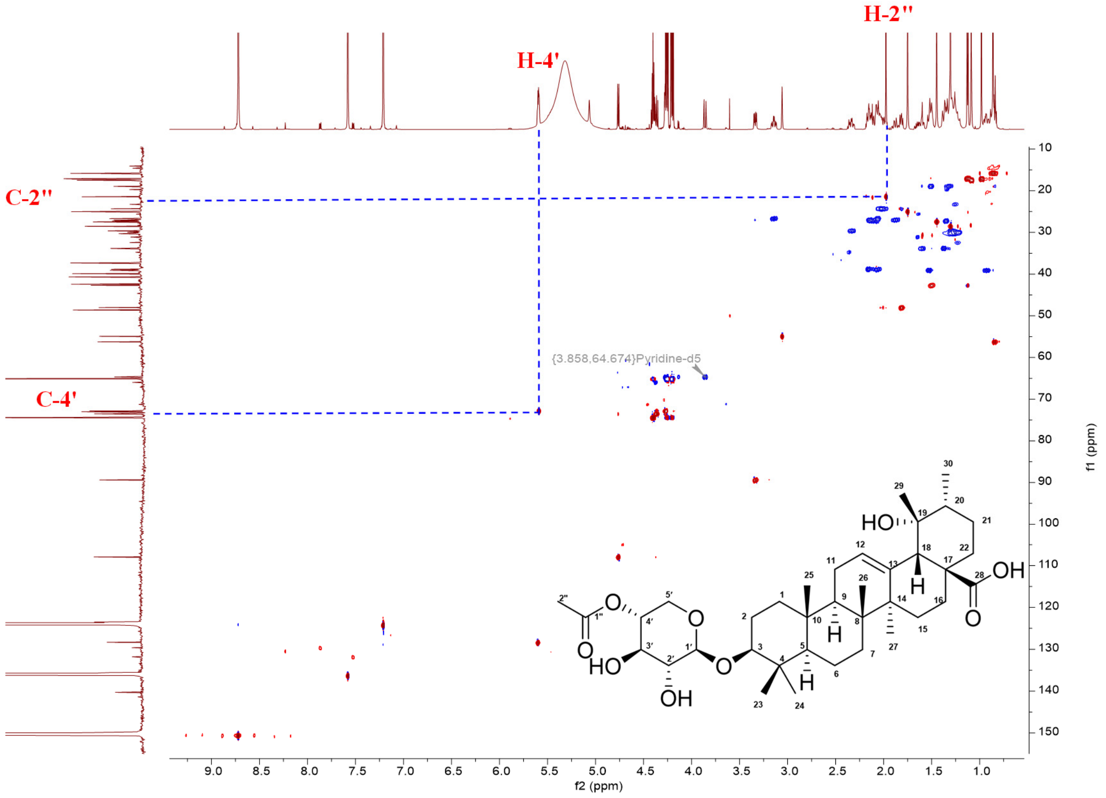

Supplementary Fig. 35 HSQC spectrum of **3b** in pyridine-*d*5 (400 MHz).

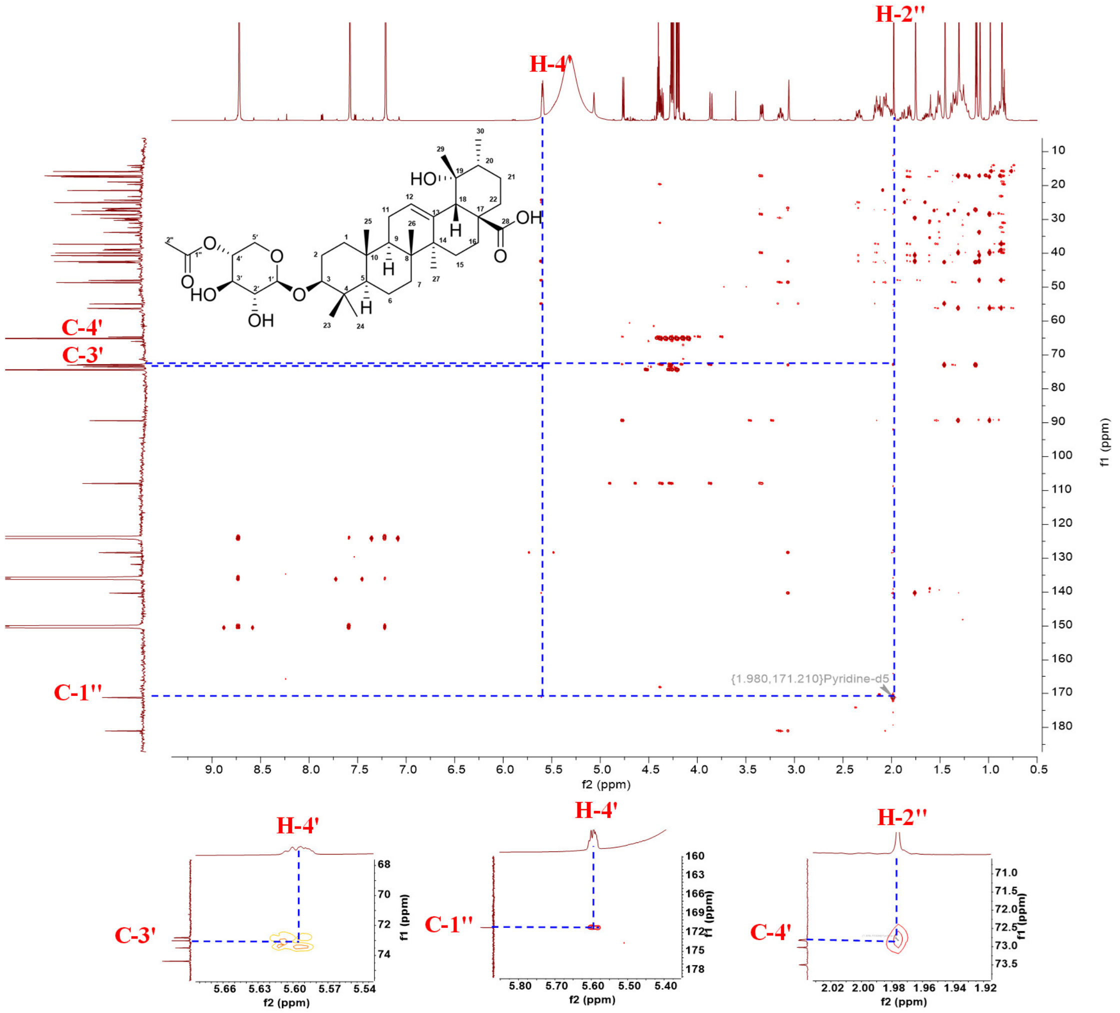

Supplementary Fig. 36 HMBC spectrum of **3b** in pyridine-*d*5 (400 MHz).

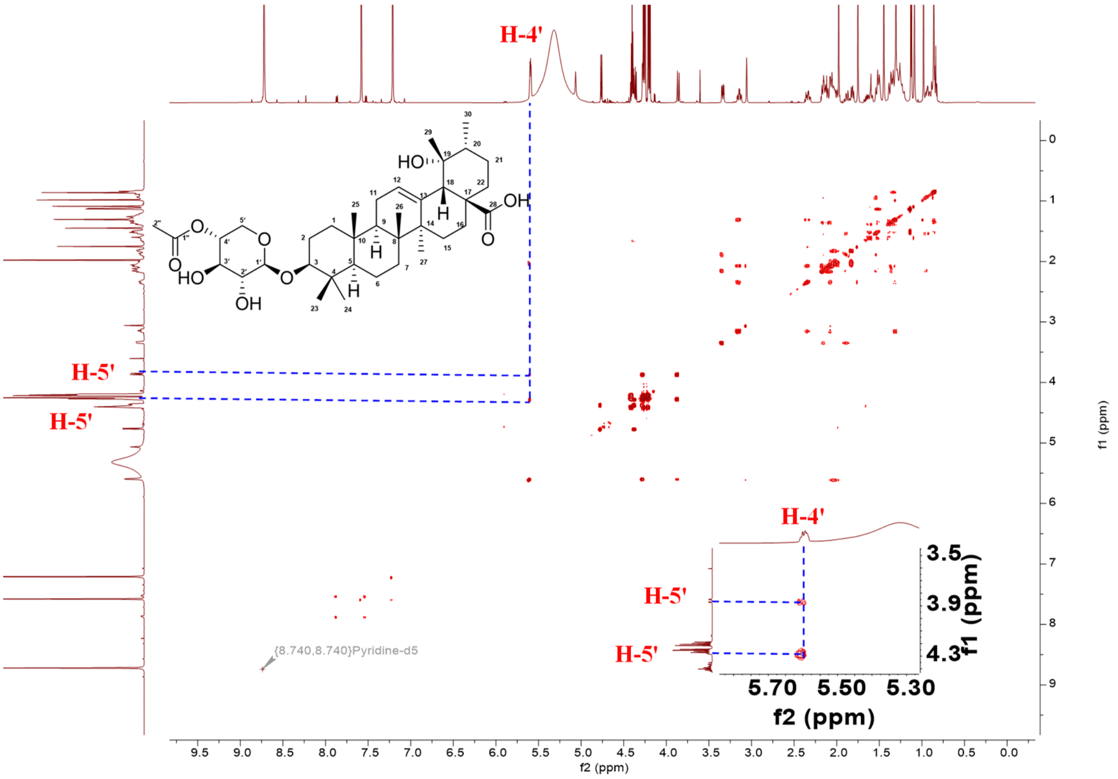

Supplementary Fig. 37 ^{1}H-^{1}H COSY spectrum of **3b** in pyridine-d_5 (400 MHz).

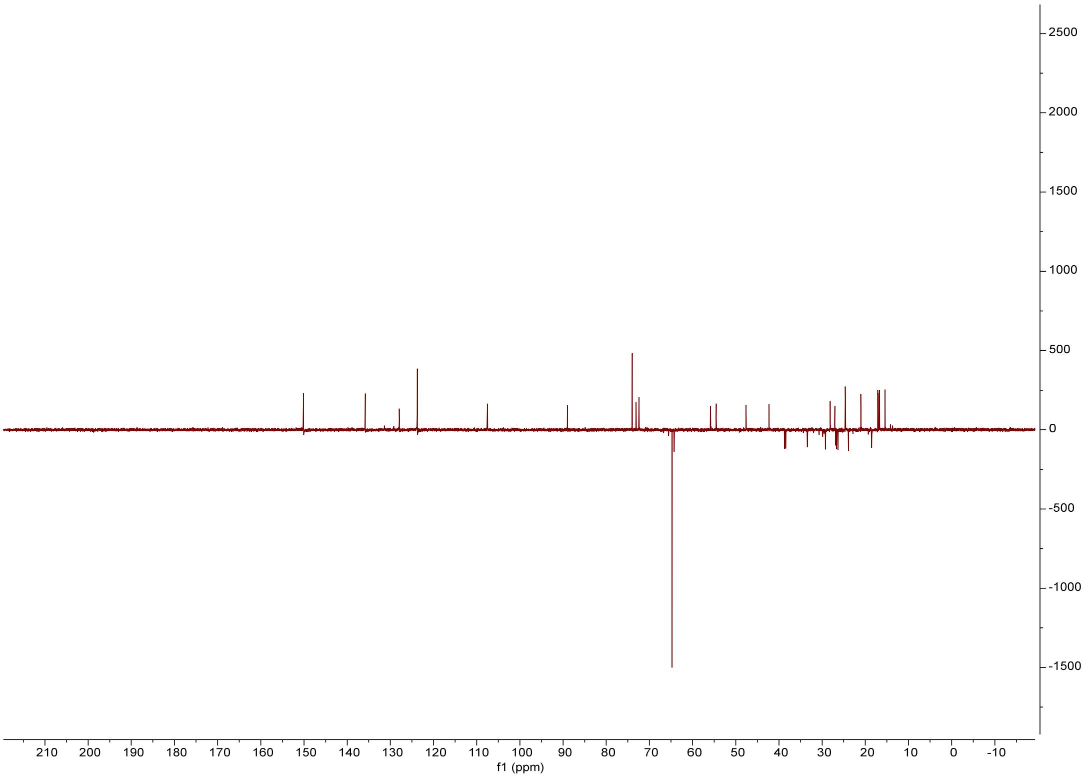

Supplementary Fig. 38 DEPT 135 spectrum of **3b** in pyridine-d_5 (400 MHz).

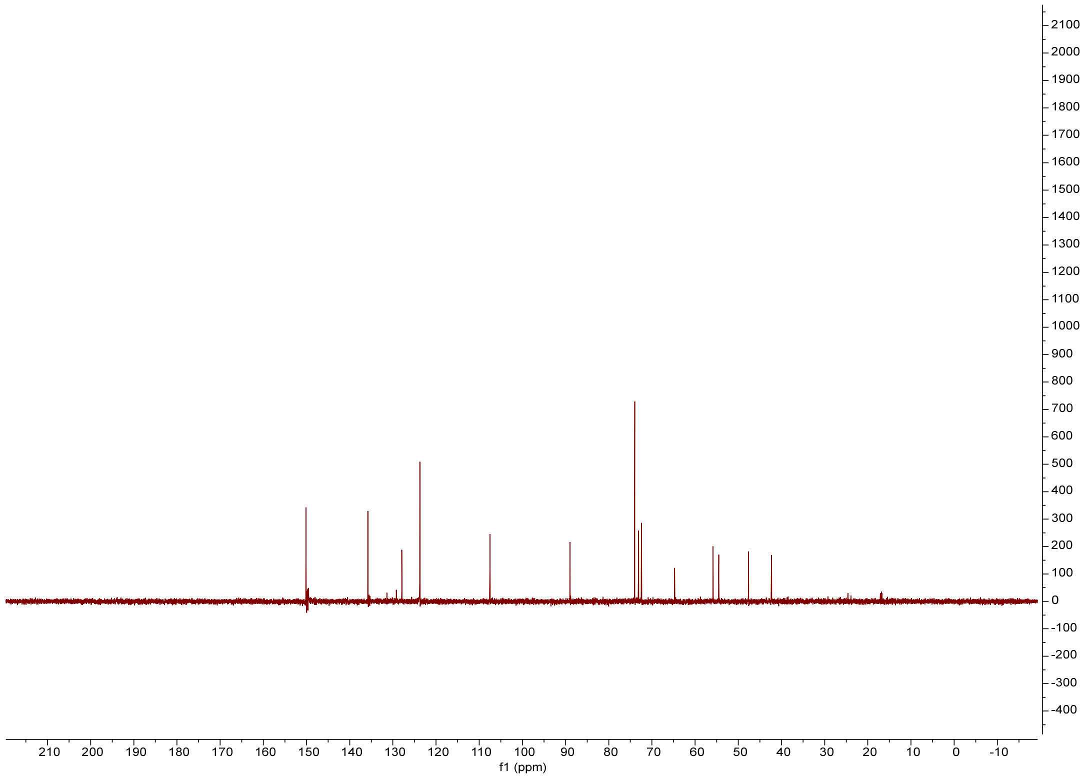

Supplementary Fig. 39 DEPT 90 spectrum of **3b** in pyridine-d_5 (400 MHz).

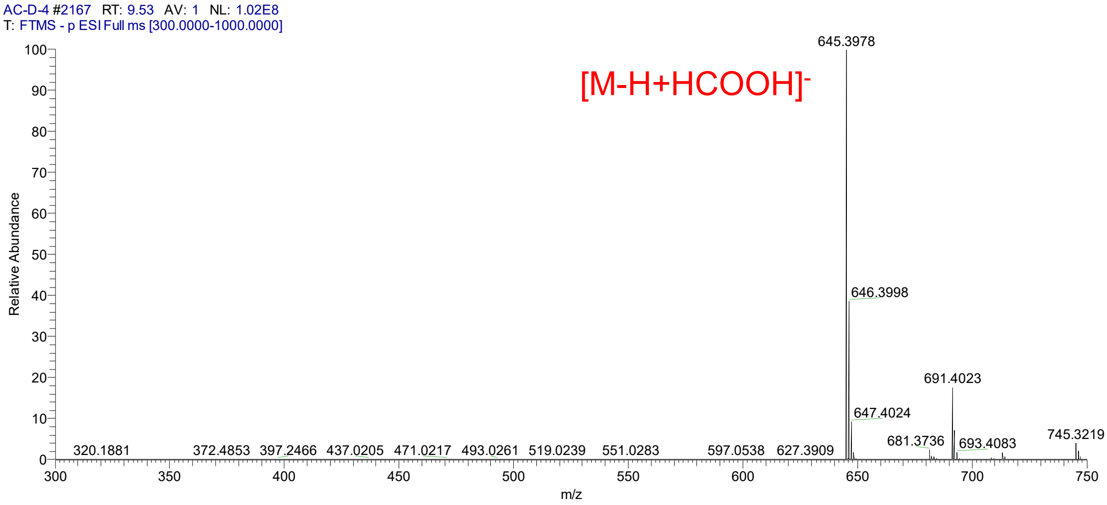

Supplementary Fig. 40 (-)ESI-HR-MS spectrum of **3b**.

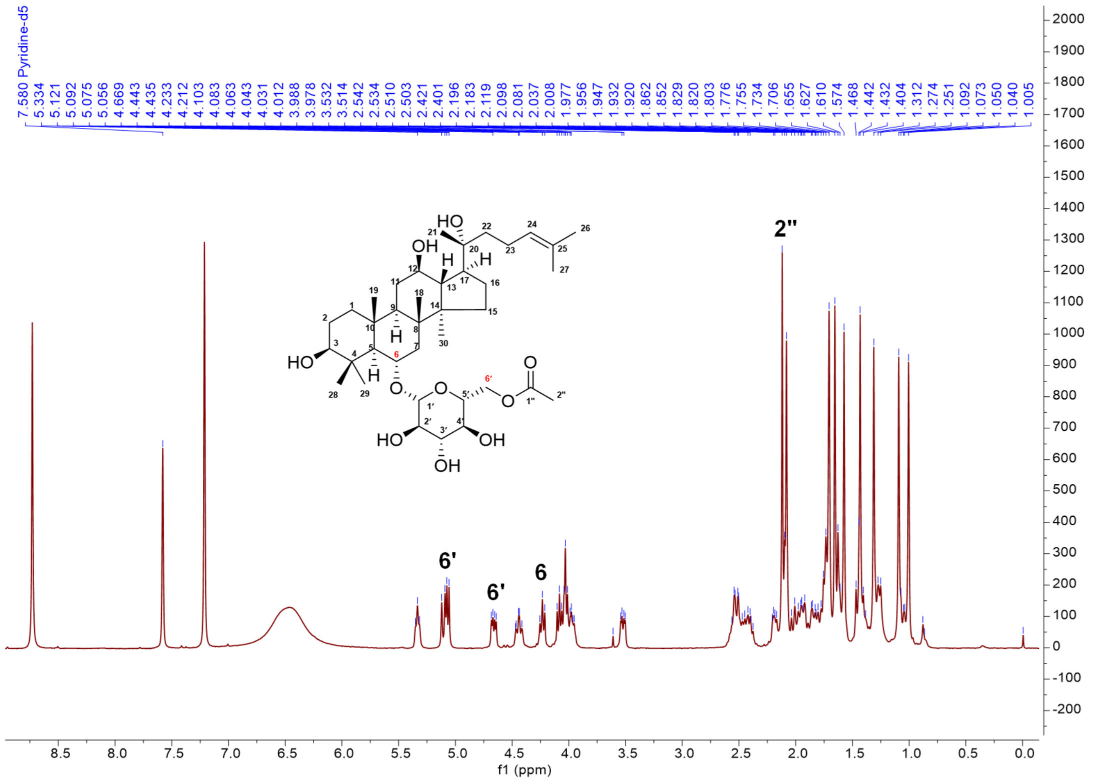

Supplementary Fig. 41 ^{1}H NMR spectrum of **6a** in pyridine-d_5 (400 MHz).

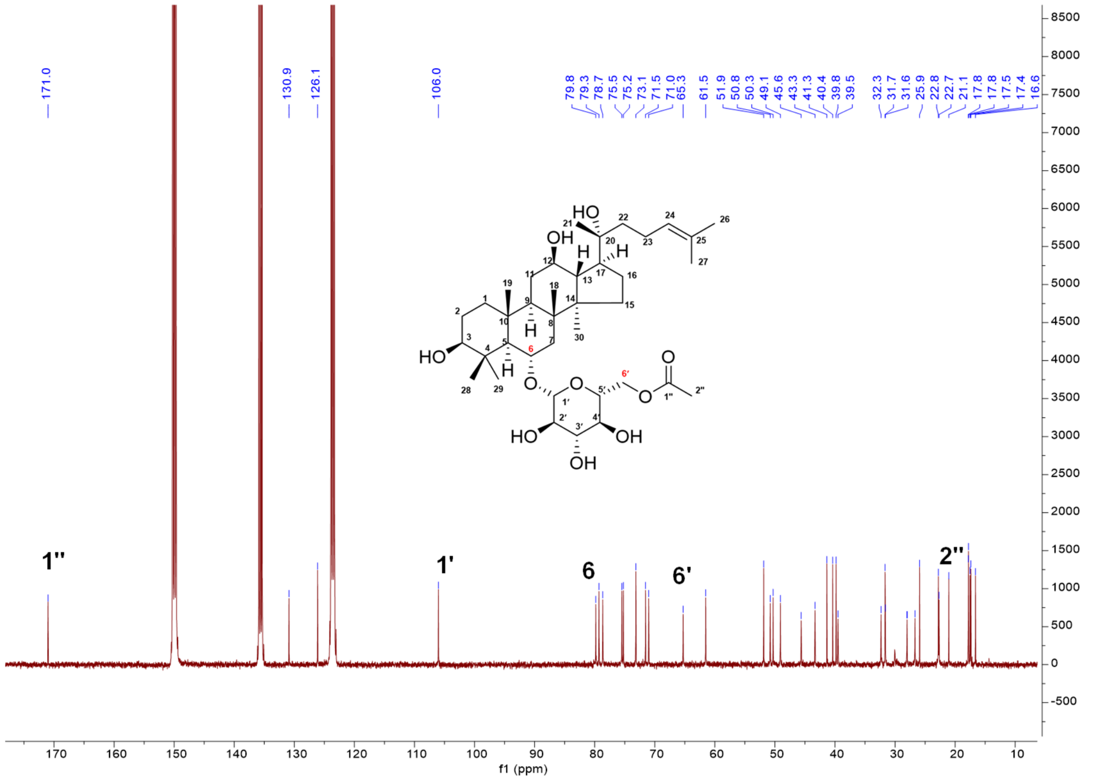

Supplementary Fig. 42 ^{13}C NMR spectrum of **6** in pyridine-d_5 (100 MHz).

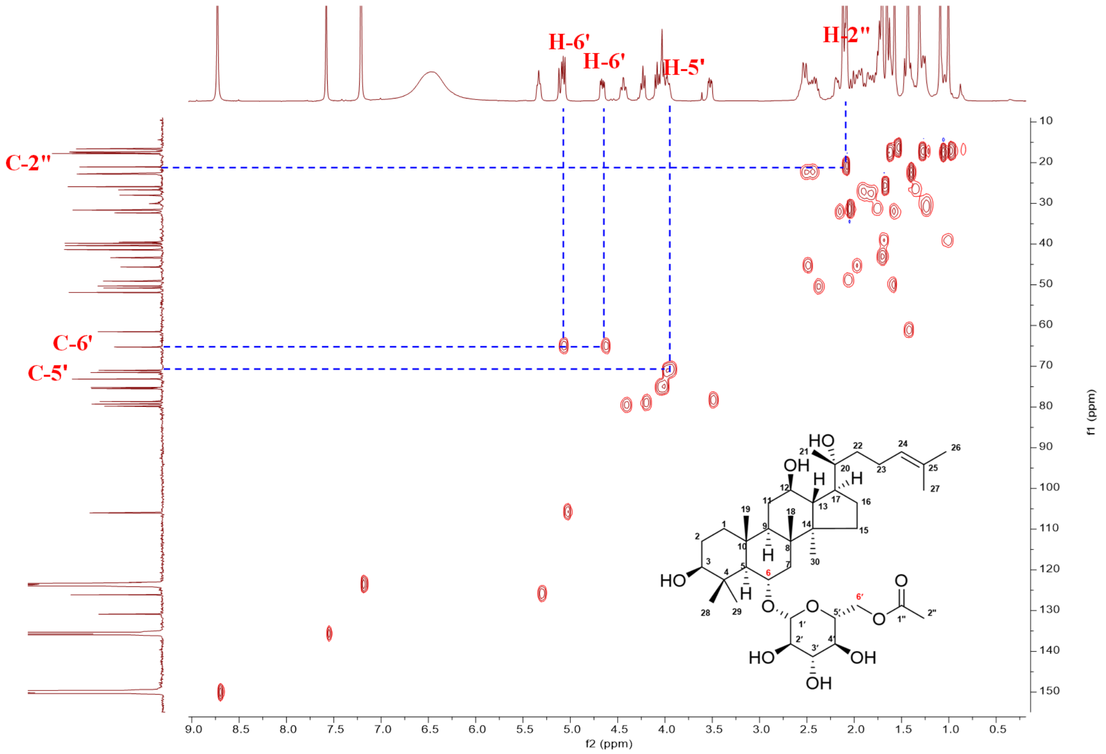

Supplementary Fig. 43 HSQC spectrum of **6a** in pyridine-d_5 (400 MHz).

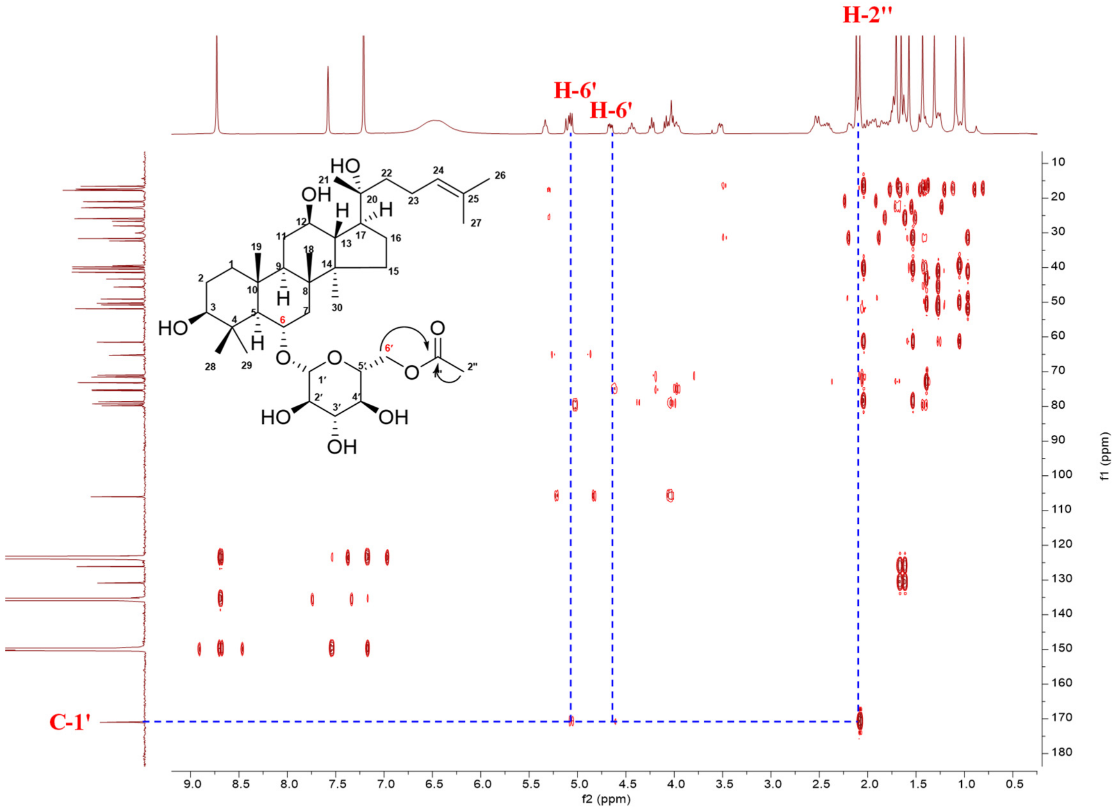

Supplementary Fig. 44 HMBC spectrum of **6a** in pyridine-d_5 (600 MHz).

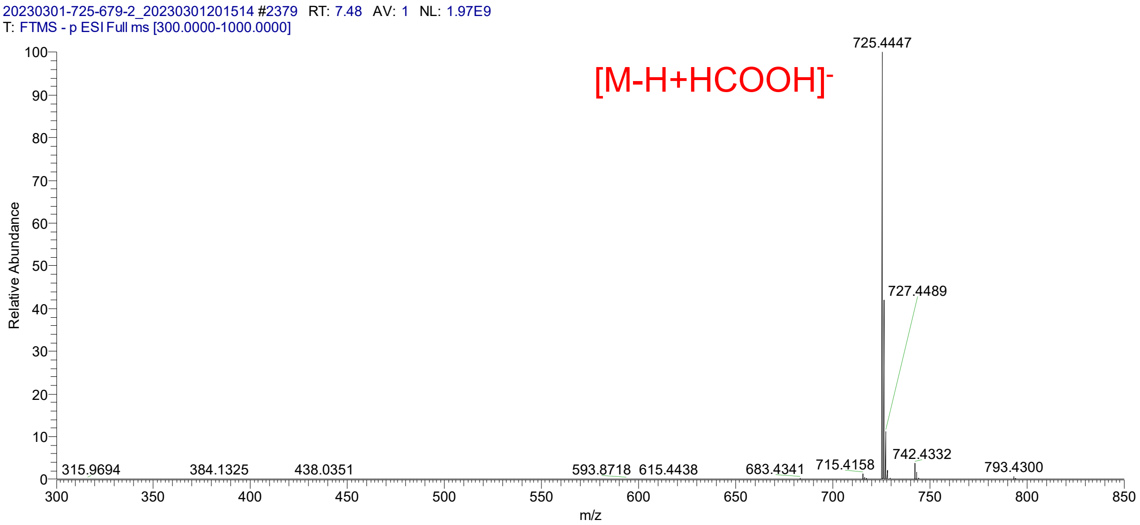

Supplementary Fig. 45 (-)ESI-HR-MS spectrum of **6a**.

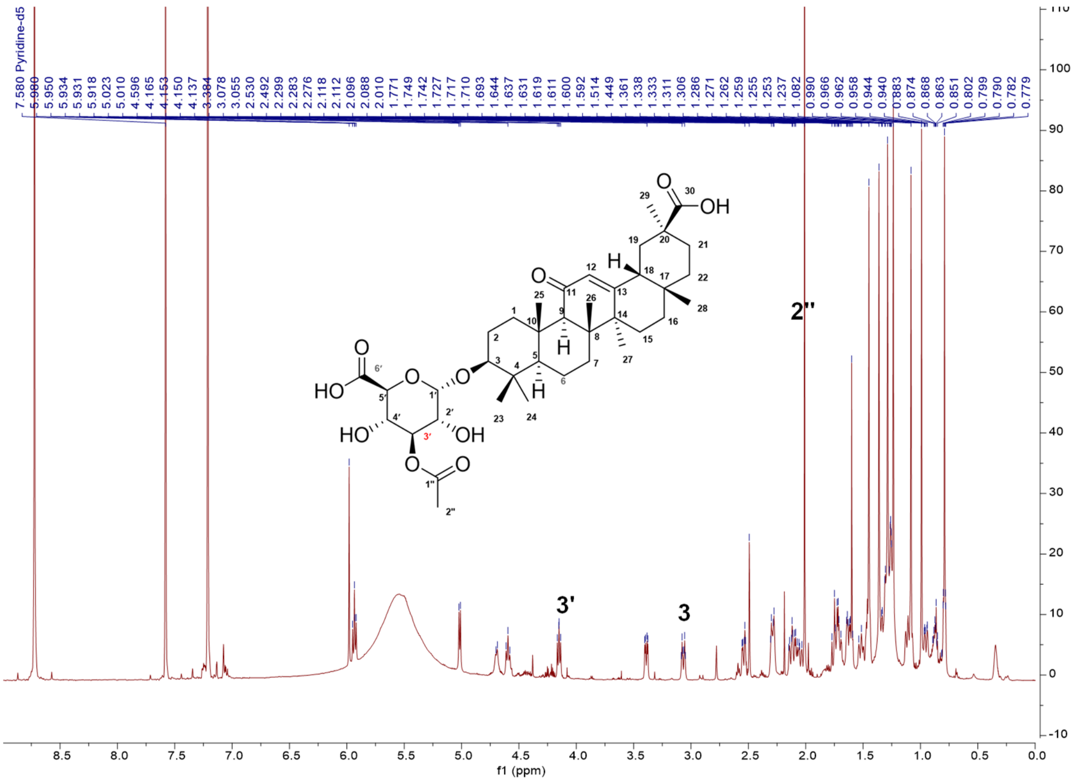

Supplementary Fig. 46 ^{1}H NMR spectrum of **7a** in pyridine-d_5 (400 MHz).

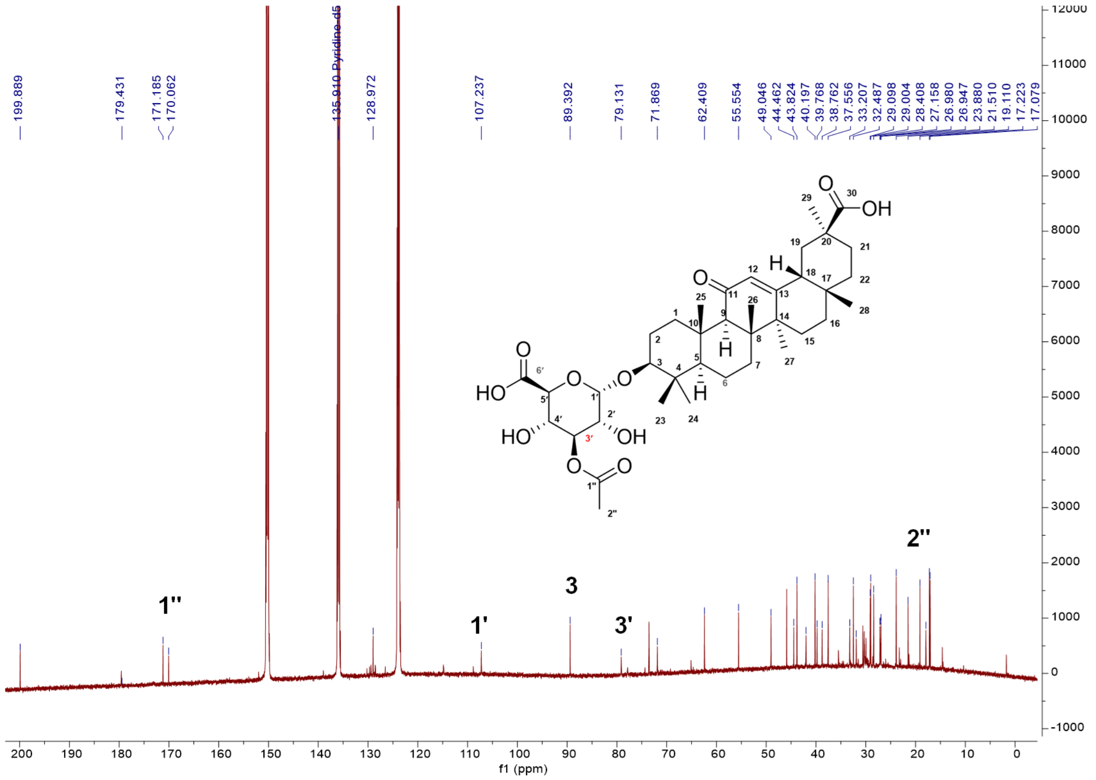

Supplementary Fig. 47 ^{13}C NMR spectrum of **7a** in pyridine-d_5 (400 MHz).

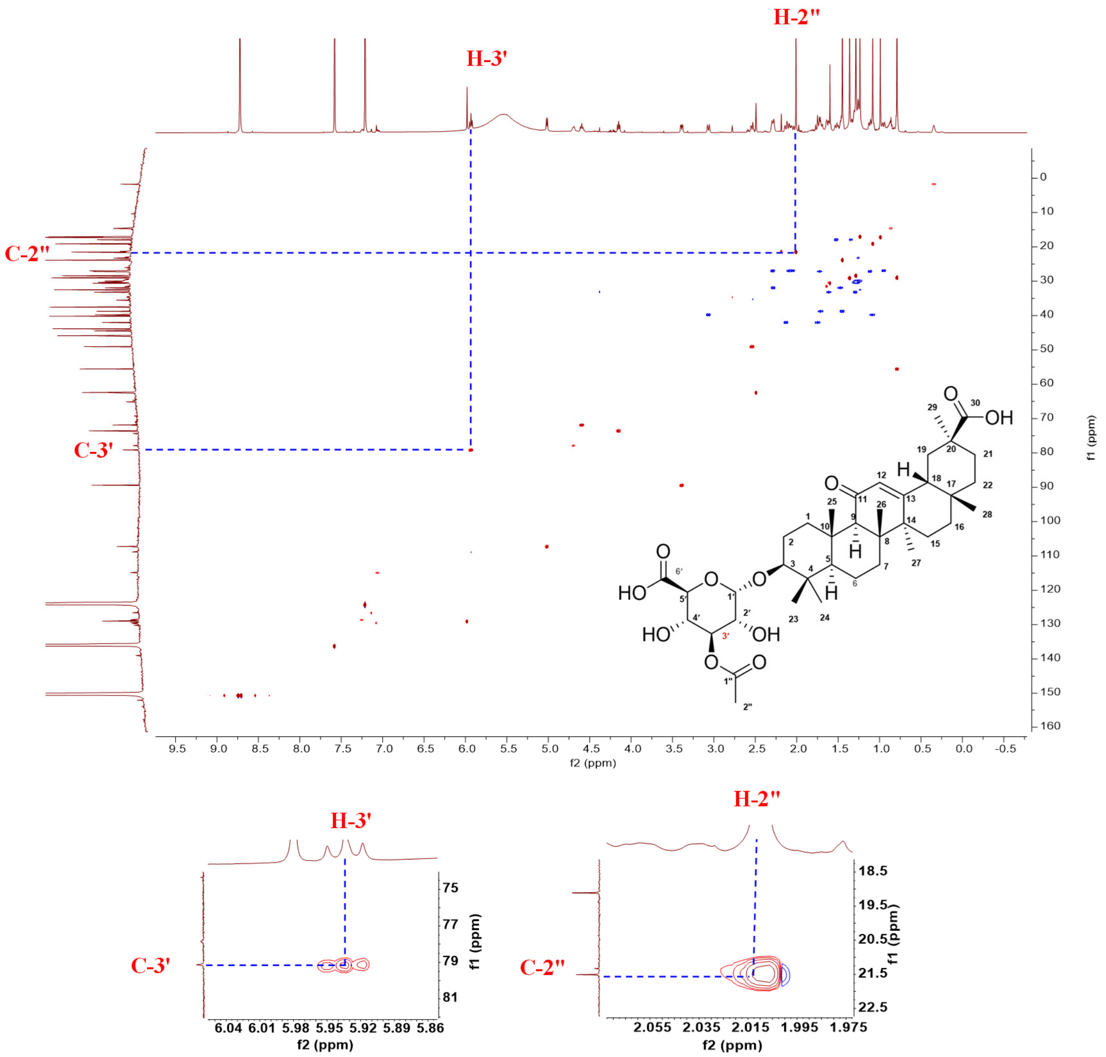

Supplementary Fig. 48 HSQC spectrum of **7a** in pyridine-d_5 (400 MHz).

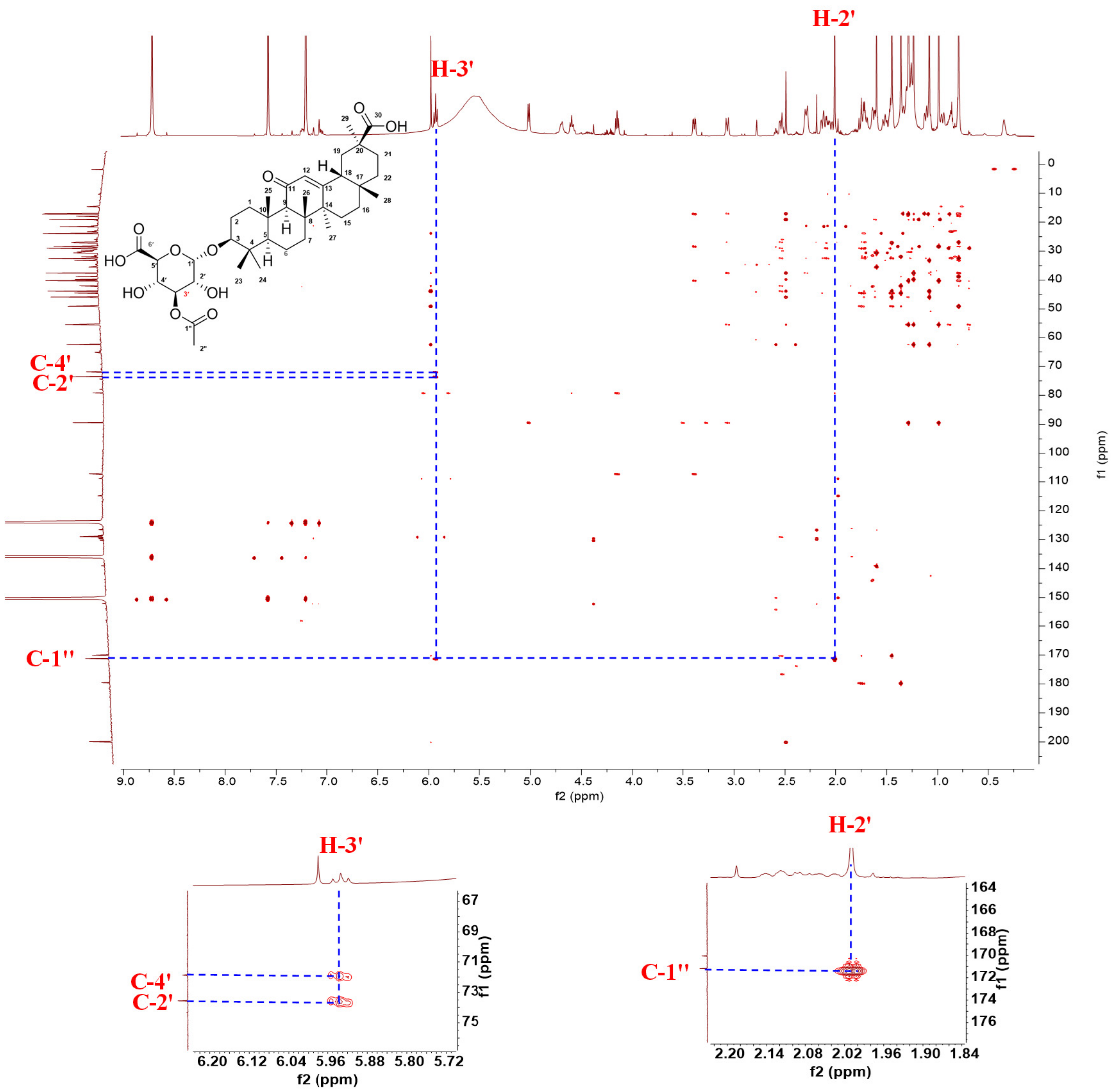

Supplementary Fig. 49 HMBC spectrum of **7a** in pyridine-d_5 (400 MHz).

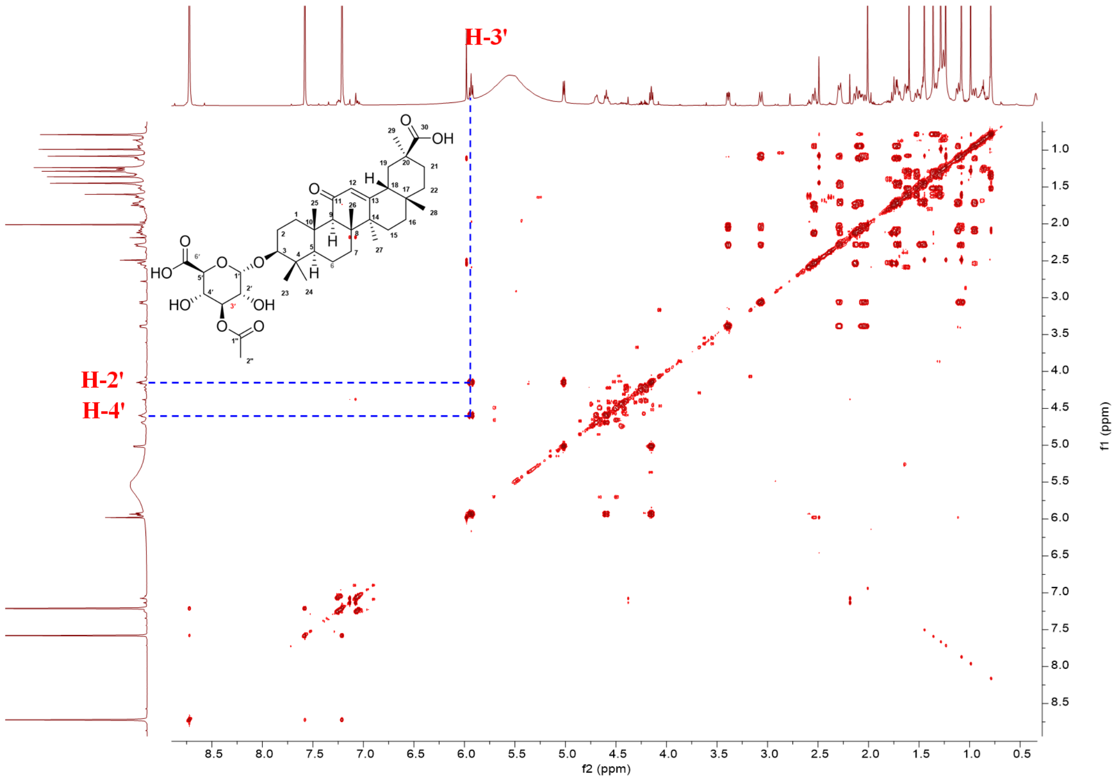

Supplementary Fig. 50 ^{1}H-^{1}H COSY spectrum of **7a** in pyridine-*d*5 (400 MHz).

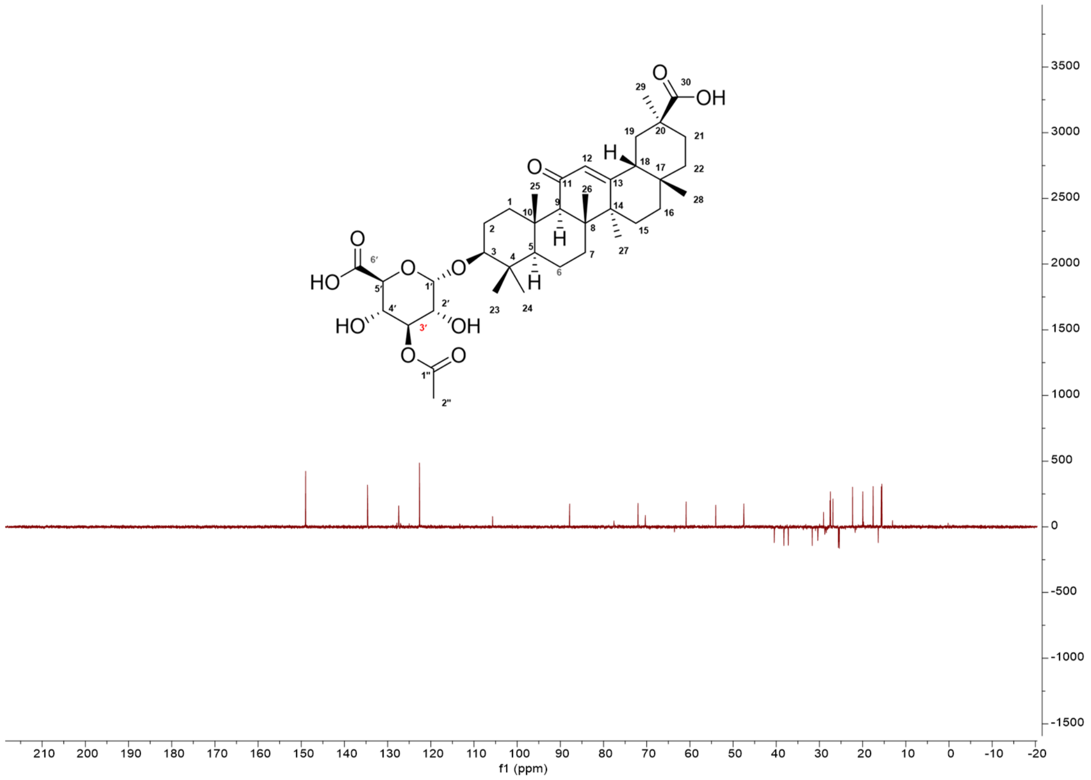

Supplementary Fig. 51 DEPT 135 spectrum of **7a** in pyridine-*d*5 (400 MHz).

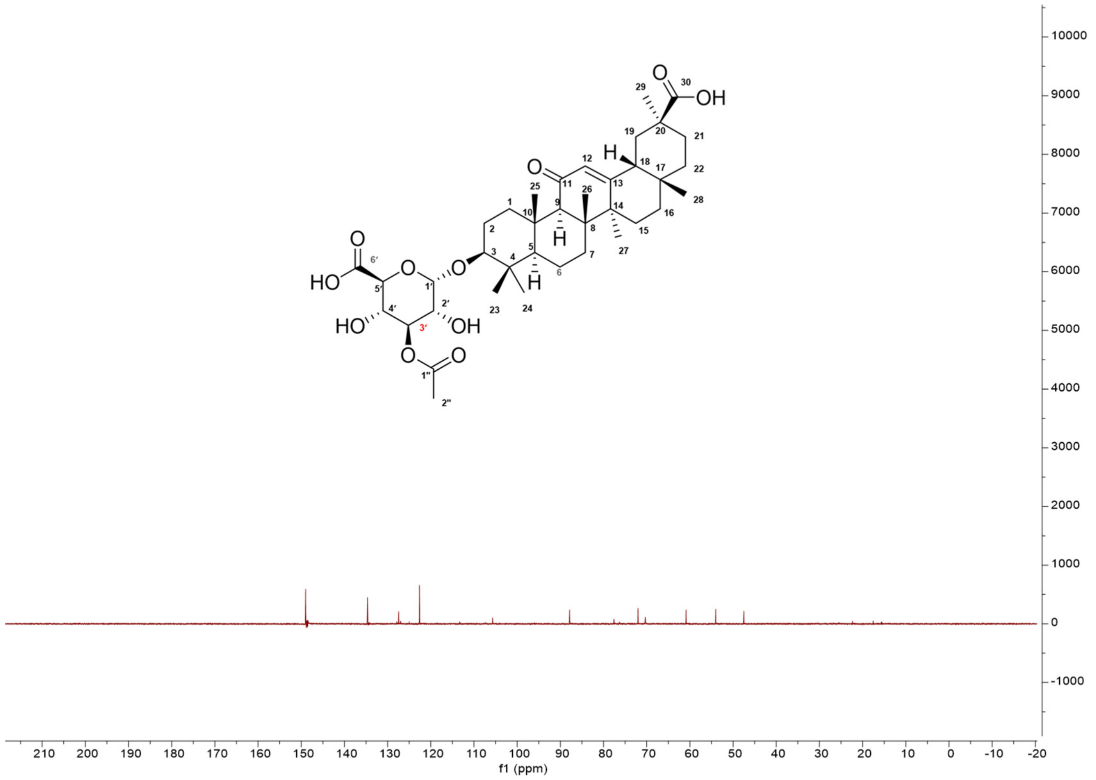

Supplementary Fig. 52 DEPT 90 spectrum of **7a** in pyridine-d_5 (400 MHz).

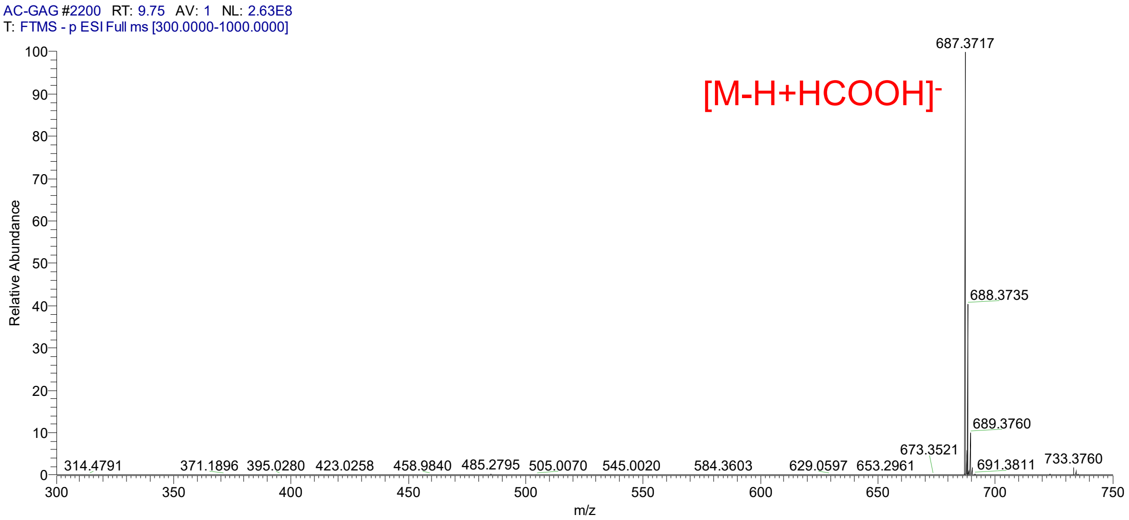

Supplementary Fig. 53 (-) ESI-HR-MS spectrum of **7a**.

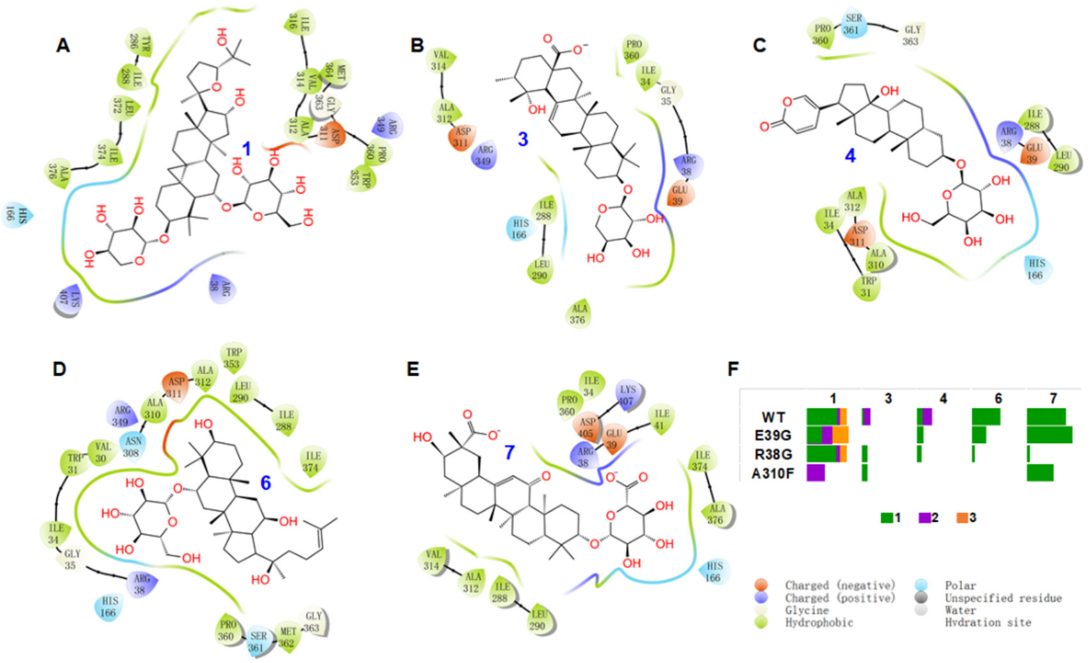

Supplementary Fig. 54 (A-E) Interaction patterns between substrates **1**/**3**/**4**/**6**/**7** and active site residues of AmAT7-3, respectively. (F) The conversion rates of different mutant lines toward substrates **1**/**3**/**4**/**6**/**7**. **a**/**b**/**c** indicated different acetylated products.

The ligand interaction was analyzed using Schrödinger with default parameters. While the amino acid residues that interact with different substrates may overlap, they might serve distinct roles with different positions of each substrate. For example, A310 was close to the sugar ring of substrates **1**/**4**, yet adjacent to the aglycone of substrates **3**/**6**/**7**. Similar observations hold for I288 and L290, which are situated close to the aglycone of substrates **1**/**6**/**7**, while close to the sugar ring of substrates **3**/**4**. These analyses indicated that structural basis for the substrate promiscuity arises from the large volume of the active pocket, which can readily accommodate sizable saponin molecules. The orientation of the saponin molecules could be determined by conserved amino acids such as R38 and E39, which establish hydrogen bonds with the sugar moiety, as supported by our functional analysis of variants E39G and R38G using substrates **1**/**3**/**4**/**6**/**7**.

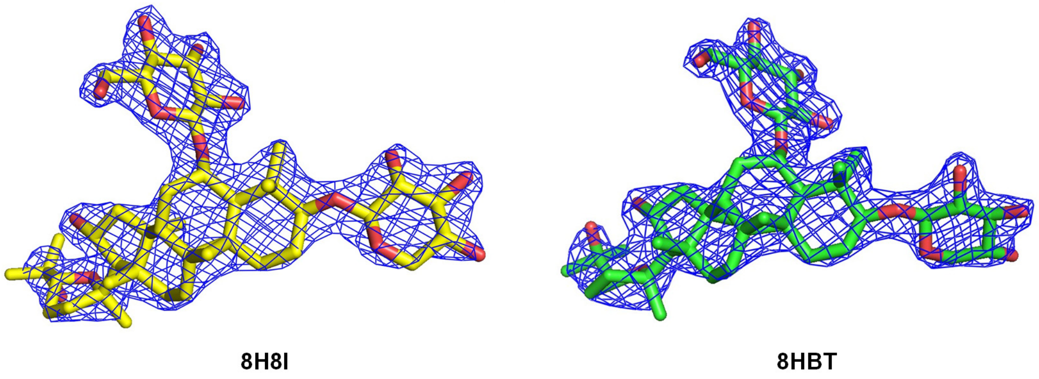

Supplementary Fig. 55 The *Fo-Fc* omit map for ligand in the crystal structures.

References

1. Zhang, X.X. et al. PoDPBT, a BAHD acyltransferase, catalyses the benzoylation in paeoniflorin biosynthesis in *Paeonia ostii*. *Plant Biotechnol J* **21**, 14-16 (2023).
2. Trott, O., & Olson, A. AutoDock Vina: improving the speed and accuracy of docking with a new scoring function, efficient optimization, and multithreading. *J Comput Chem* **31**, 455–461 (2010).
3. Pettersen, E., Goddard, T., Huang, C., Couch, G., Greenblatt, D., Meng, E., & Ferrin, T. UCSF Chimera--a visualization system for exploratory research and analysis. *J Comput Chem* **25**, 1605–1612 (2004).
4. Tian L., Feiwu C., Multiwfn: a multifunctional wavefunction analyzer. *J Comput Chem* **33**, 580-592 (2012).
5. Sadiq, S., & Coveney, P. Computing the role of near attack conformations in an enzyme-catalyzed nucleophilic bimolecular reaction. *J Chem Theory Comput* **11**, 316–324 (2015).

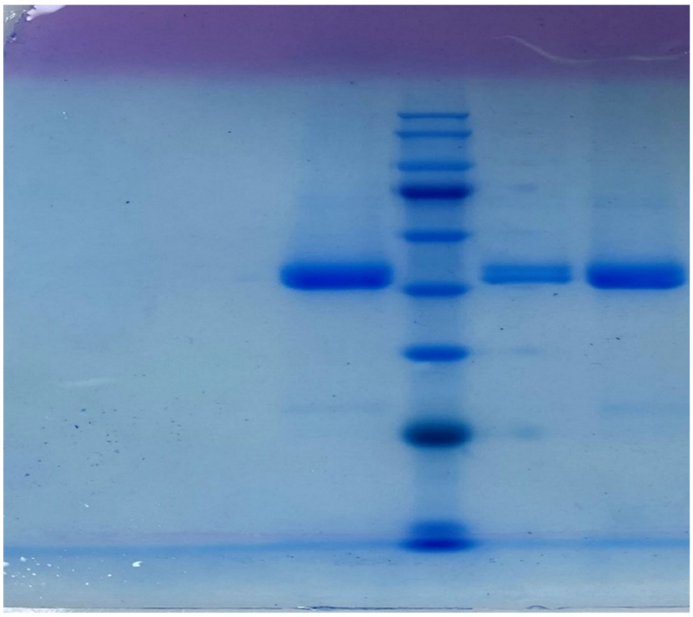

Source Data of SDS-PAGE of AmAT7-3, AmAT7-3_{A310G}, and AmAT7-3_{A310W} proteins in Supplementary Fig. 9.
