# Supplementary material for: Characterization and structure-based protein engineering of a regiospecific saponin acetyltransferase from Astragalus membranaceus
Source: Nat Commun. 2023 Sep 25;14:5969. doi: 10.1038/s41467-023-41599-7 (PMC10519980; doi:10.1038/s41467-023-41599-7)
Supplement: Supplementary file 2 — Reporting Summary [file 41467_2023_41599_MOESM2_ESM.pdf]

nature portfolio

Corresponding author(s): Xue Qiao

Last updated by author(s): Sep 8, 2023

Reporting Summary

Nature Portfolio wishes to improve the reproducibility of the work that we publish. This form provides structure for consistency and transparency in reporting. For further information on Nature Portfolio policies, see our Editorial Policies and the Editorial Policy Checklist.

Statistics

For all statistical analyses, confirm that the following items are present in the figure legend, table legend, main text, or Methods section.

n/a	Confirmed	
☐	☒	The exact sample size (*n*) for each experimental group/condition, given as a discrete number and unit of measurement
☐	☒	A statement on whether measurements were taken from distinct samples or whether the same sample was measured repeatedly
☒	☐	The statistical test(s) used AND whether they are one- or two-sided<br>*Only common tests should be described solely by name; describe more complex techniques in the Methods section.*
☐	☒	A description of all covariates tested
☒	☐	A description of any assumptions or corrections, such as tests of normality and adjustment for multiple comparisons
☐	☒	A full description of the statistical parameters including central tendency (e.g. means) or other basic estimates (e.g. regression coefficient) AND variation (e.g. standard deviation) or associated estimates of uncertainty (e.g. confidence intervals)
☒	☐	For null hypothesis testing, the test statistic (e.g. *F*, *t*, *r*) with confidence intervals, effect sizes, degrees of freedom and *P* value noted<br>*Give P values as exact values whenever suitable.*
☒	☐	For Bayesian analysis, information on the choice of priors and Markov chain Monte Carlo settings
☒	☐	For hierarchical and complex designs, identification of the appropriate level for tests and full reporting of outcomes
☒	☐	Estimates of effect sizes (e.g. Cohen's *d*, Pearson's *r*), indicating how they were calculated

Our web collection on statistics for biologists contains articles on many of the points above.

Software and code

Policy information about availability of computer code

Data collection	The pka value of residue was predicted by PROPKA (http://server.poissonboltzmann.org/pdb2pqr). Amber 18 was used for molecular dynamics simulations. QM/MM was performed with ChemShell 3.5.0, combining turbomole (7.5) for the QM region. All dockings were performed using the AutoDock Vina (1.2.3). The collection and analysis of mass spectrometry data is completed using Xcalibur software.
Data analysis	OriginPro (8) was used for the analysis of QM data. Analyses of molecular dynamics simulation trajectories were carried out uusing standard tools of amber, VMD(1.9.4) and Multiwfn(3.8). The collection and analysis of mass spectrometry data is completed using Xcalibur software.

For manuscripts utilizing custom algorithms or software that are central to the research but not yet described in published literature, software must be made available to editors and reviewers. We strongly encourage code deposition in a community repository (e.g. GitHub). See the Nature Portfolio guidelines for submitting code & software for further information.

Data

Policy information about availability of data

All manuscripts must include a data availability statement. This statement should provide the following information, where applicable:

- Accession codes, unique identifiers, or web links for publicly available datasets
- A description of any restrictions on data availability
- For clinical datasets or third party data, please ensure that the statement adheres to our policy

The sequences mentioned in this study have been deposited in NCBI, the accession number of AmAT7-3 is ON804888; the accession number of AmAT8-17 is OQ915518. The crystal structures obtained in this study have been deposited in the Protein Data Bank: AmAT7-3/astragaloside IV (PDB number 8H8I),

AmAT7-3A310G/astragaloside IV (PDB number 8HBT). Source data are provided with this paper.

Research involving human participants, their data, or biological material

Policy information about studies with human participants or human data. See also policy information about sex, gender (identity/presentation), and sexual orientation and race, ethnicity and racism.

Reporting on sex and gender	N/A.
Reporting on race, ethnicity, or other socially relevant groupings	N/A.
Population characteristics	N/A.
Recruitment	N/A.
Ethics oversight	N/A.

Note that full information on the approval of the study protocol must also be provided in the manuscript.

Field-specific reporting

Please select the one below that is the best fit for your research. If you are not sure, read the appropriate sections before making your selection.

- [x] Life sciences
- [ ] Behavioural & social sciences
- [ ] Ecological, evolutionary & environmental sciences

For a reference copy of the document with all sections, see nature.com/documents/nr-reporting-summary-flat.pdf

Life sciences study design

All studies must disclose on these points even when the disclosure is negative.

Sample size	No sample size calculation was performed since the work mainly reported results from enzyme catalytic reactions. Data for AmAT7-3 biochemical analyses are from n = 2 or 3 independent measurements, which were normally used to reflect the errors in enzyme catalytic experiments. Data for mutant screening and substrate promiscuity screening are n = 1, which was typically used in catalytic function screening experiments.
Data exclusions	No data were excluded.
Replication	For AmAT7-3 biochemical analyses, 2 or 3 biological replicates were used as depicted in main text, methods, or figure captions. For the Subcellular localization experiment, 3 technical replicates were used as mentioned in the figure caption. All experiments could be replicated and results from different replicates were consistent.
Randomization	Randomization is not relevant to our study. Samples from biocatalytic reactions were analyzed in a random manner.
Blinding	Blinding is not relevant to our study.

Reporting for specific materials, systems and methods

We require information from authors about some types of materials, experimental systems and methods used in many studies. Here, indicate whether each material, system or method listed is relevant to your study. If you are not sure if a list item applies to your research, read the appropriate section before selecting a response.

Materials & experimental systems

n/a	Involved in the study	
x		Antibodies
x		Eukaryotic cell lines
x		Palaeontology and archaeology
x		Animals and other organisms
x		Clinical data
x		Dual use research of concern
	x	Plants

Methods

n/a	Involved in the study	
x		ChIP-seq
x		Flow cytometry
x		MRI-based neuroimaging

Dual use research of concern

Policy information about dual use research of concern

Hazards

Could the accidental, deliberate or reckless misuse of agents or technologies generated in the work, or the application of information presented in the manuscript, pose a threat to:

No	Yes	
[x]	[ ]	Public health
[x]	[ ]	National security
[x]	[ ]	Crops and/or livestock
[x]	[ ]	Ecosystems
[x]	[ ]	Any other significant area

Experiments of concern

Does the work involve any of these experiments of concern:

No	Yes	
[x]	[ ]	Demonstrate how to render a vaccine ineffective
[x]	[ ]	Confer resistance to therapeutically useful antibiotics or antiviral agents
[x]	[ ]	Enhance the virulence of a pathogen or render a nonpathogen virulent
[x]	[ ]	Increase transmissibility of a pathogen
[x]	[ ]	Alter the host range of a pathogen
[x]	[ ]	Enable evasion of diagnostic/detection modalities
[x]	[ ]	Enable the weaponization of a biological agent or toxin
[x]	[ ]	Any other potentially harmful combination of experiments and agents
