# Supplementary material for: Characterization and structure-based protein engineering of a regiospecific saponin acetyltransferase from Astragalus membranaceus
Source: Nat Commun. 2023 Sep 25;14:5969. doi: 10.1038/s41467-023-41599-7 (PMC10519980; doi:10.1038/s41467-023-41599-7)
Supplement: Supplementary file 3 — Description of Additional Supplementary Files [file 41467_2023_41599_MOESM3_ESM.pdf]

Title: Supplementary Data 1
Description: Summary of acyltransferases used for phylogenetic analysis.

Title: Supplementary Data 2
Description: Primers for site-directed mutagenesis experiments.’.
