# Supplementary material for: Characterization and structure-based protein engineering of a regiospecific saponin acetyltransferase from Astragalus membranaceus
Source: Nat Commun. 2023 Sep 25;14:5969. doi: 10.1038/s41467-023-41599-7 (PMC10519980; doi:10.1038/s41467-023-41599-7)
Supplement: Supplementary file 7 — Source Data [file 41467_2023_41599_MOESM7_ESM.zip › Source Data/Inventory of Source Data.docx]

**Inventory of Source Data**

| Folder | File name | Descriptions |
| --- | --- | --- |
| Source Data | Source Data-R2 | The source data of the figures in both manuscript and Supplementary Information, each sheet has been named with its figure number. |
| Source Data | Optimized Cartesian coordinates of stationary points.txt | The atomic coordinates of the optimized computational models. |
| Source Data/  QMMM_optimized_RC | QMMM_optimized_RC_A310G | The QM/MM-optimized structures of RC for A310G. |
|  | QMMM_optimized_RC_A310W | The QM/MM-optimized structures of RC for A310W. |
|  | QMMM_optimized_RC_WT_conformation_1 | The QM/MM-optimized structures of RC for conformation 1 of AmAT7-3. |
|  | QMMM_optimized_RC_WT_conformation_2 | The QM/MM-optimized structures of RC for conformation 2 of AmAT7-3. |
| Source Data/  MD configurations | A310F-final.txt | The final configurations of MD simulations for A310F. Others are named using the same regulation. |
|  | A310F-start.txt | The start configurations of MD simulations for A310F. Others are named using the same regulation. |
